# Structural basis for the activity regulation of a potassium channel AKT1 from Arabidopsis

**DOI:** 10.1038/s41467-022-33420-8

**Published:** 2022-09-27

**Authors:** Yaming Lu, Miao Yu, Yutian Jia, Fan Yang, Yanming Zhang, Xia Xu, Xiaomin Li, Fan Yang, Jianlin Lei, Yi Wang, Guanghui Yang

**Affiliations:** 1 State Key Laboratory for Agrobiotechnology, Frontiers Science Center for Molecular Design Breeding, College of Biological Sciences, China Agricultural University, Beijing, China; 2 State Key Laboratory of Plant Physiology and Biochemistry, College of Biological Sciences, China Agricultural University, Beijing, China; 3 Technology Center for Protein Sciences, Ministry of Education Key Laboratory of Protein Sciences, School of Life Sciences, Tsinghua University, Beijing, China

**Keywords:** Potassium channels, Cryoelectron microscopy, Membrane proteins, Permeation and transport

## Abstract

The voltage-gated potassium channel AKT1 is responsible for primary K^+^ uptake in *Arabidopsis* roots. AKT1 is functionally activated through phosphorylation and negatively regulated by a potassium channel α-subunit AtKC1. However, the molecular basis for the modulation mechanism remains unclear. Here we report the structures of AKT1, phosphorylated-AKT1, a constitutively-active variant, and AKT1-AtKC1 complex. AKT1 is assembled in 2-fold symmetry at the cytoplasmic domain. Such organization appears to sterically hinder the reorientation of C-linkers during ion permeation. Phosphorylated-AKT1 adopts an alternate 4-fold symmetric conformation at cytoplasmic domain, which indicates conformational changes associated with symmetry switch during channel activation. To corroborate this finding, we perform structure-guided mutagenesis to disrupt the dimeric interface and identify a constitutively-active variant Asp379Ala mediates K^+^ permeation independently of phosphorylation. This variant predominantly adopts a 4-fold symmetric conformation. Furthermore, the AKT1-AtKC1 complex assembles in 2-fold symmetry. Together, our work reveals structural insight into the regulatory mechanism for AKT1.

## Abstract (web summary)

*Arabidopsis thaliana* potassium channel AKT1 is responsible for primary K + uptake from soil, which is functionally activated through phosphorylation and negatively regulated by an α-subunit AtKC1. Here, the authors report the structures of AKT1 at different states, revealing a 2- fold to 4-fold symmetry switch at cytoplasmic domain associated with AKT1 activity regulation.

## Introduction

AKT1 is the first characterized hyperpolarization-activated voltage-dependent K^+^ channel in *Arabidopsis thaliana*, which belongs to the Shaker K^+^ channel family^1–3^. AKT1 is a founding member of plant K^+^ channels^1–3^. Physiologically, AKT1 and a K^+^ transporter HAK5 serve as the major components for primary K^+^ uptake from soil^2,4^. This process depends on phosphorylation in response to the change of K^+^ concentration or stress^4–9^. In the phosphorylation cascade, the calcineurin B-like protein 1 or 9 (CBL1/9) activates CBL-interacting serine/threonine-protein kinase 23 (CIPK23). The activated CIPK23 directly phosphorylates AKT1 to boost K^+^ influx^5,10^. Different from most K^+^ channels, AKT1 is electrically silent if expressed alone in *Xenopus* oocytes^5^. In the absence of either CBL1/9 or CIPK23, no currents can be recorded by AKT1-mediated K^+^ influx in *Xenopus* oocytes and the protoplast of root cells^5,11^.

AKT1 forms either homo-tetrameric channel with four identical pore-forming subunits or hetero-tetrameric channel with a potassium channel α-subunit AtKC1^12,13^. AtKC1 does not transduce K^+^ when expressed alone, but it inhibits the activity of AKT1 by forming an AKT1-AtKC1 complex^12,14^. The activity regulation of AKT1 by AtKC1 is under the control of the phosphorylation cascade as well. CBL1/9 and CIPK23 are required for the function of AKT1 in either homo- or hetero-tetramers^12,13,15^. However, the regulation mechanism of AKT1 remains unclear.

In this work, we report AKT1 structures in different conformations that correlate with its distinct channel activities. The C-linkers of AKT1 undergo pronounced conformational rearrangement in different states, which sheds lights on the structural basis for activity regulation of AKT1.

## Results

### Assembly of AKT1 in 2-fold symmetry

**Fig. 1:**
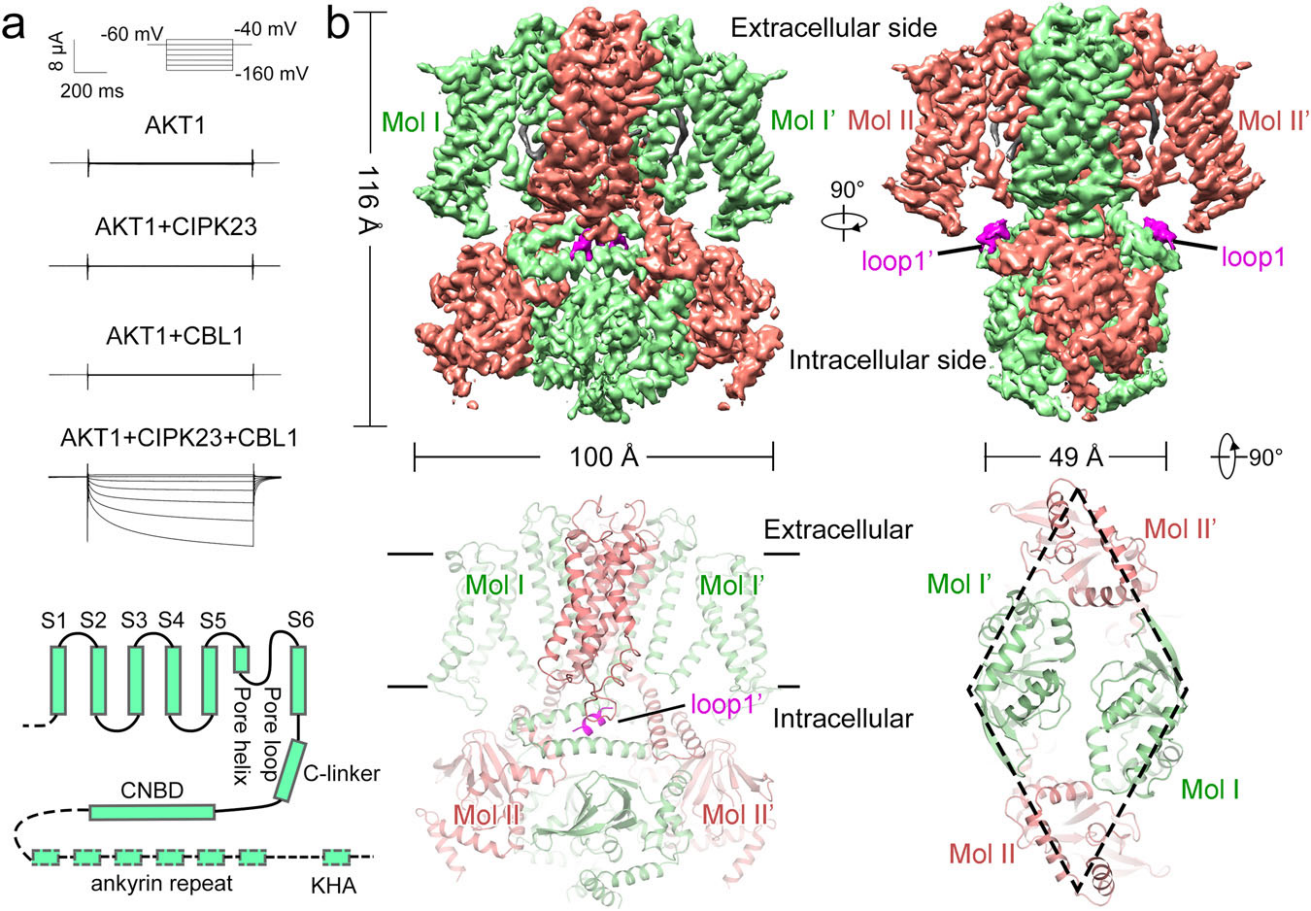
Functional validation and structural determination of the potassium channel AKT1 from *Arabidopsis*. **a** Whole-cell current trace of the AKT1 recorded in *Xenopus* oocytes. AKT1-mediated inward currents is dependent on the presence of both CIPK23 and CBL1. A diagram depicting the major domains in a AKT1 subunit is shown. **b** The atomic structure of AKT1 exhibits 2-fold symmetry viewed from the cytoplasmic side. Subunits sharing the same conformation are labeled in the same color. The four subunits are named as Mol I, Mol I’, Mol II, and Mol II’. The loops1/1’ are colored in magenta. Electrophysiological experiments were repeated using three different batches of oocytes with similar results. Source data are provided as a Source Data file.

The channel activity of AKT1 was examined by a two-electrode voltage clamp recording system in *Xenopus* oocytes. In agreement with previous studies^5^, the currents of AKT1 generated by K^+^ influx requires the presence of both CIPK23 and CBL1 (Fig. 1a). To investigate the structure-function relationship of AKT1, the protein was purified to homogeneity for cryo-electron microscopy (cryo-EM) analysis (Supplementary Fig. 1). In total 1017 micrographs were recorded using a K3 Summit detector mounted on a Titan Krios microscope, yielding 349,544 particles (Supplementary Fig. 1a). The extracted particles were applied to two-dimensional (2D) classifications. Selected particles were used to generate an initial model and then subjected to three-dimensional (3D) classifications. The model of 3D classification displayed a 2-fold but not 4-fold symmetric assembly at the cytoplasmic domain (CPD) (Supplementary Fig. 1b, c). This is consistent with the images of several bottom-viewed classes from 2D classification (Supplementary Fig. 1a). In total, 181,714 particles were used for auto-refinement, yielding a final reconstruction of AKT1 map at an overall resolution of 3.4 Å (Supplementary Fig. 1d and Supplementary Table 1). The widths of the CPDs are estimated to ~49 and 100 Å in two perpendicular side views (Fig. 1b).

On the basis of the EM densities, residues 48–510 were de novo built for each subunit (Fig. 1 and Supplementary Fig. 2a, b and Supplementary Table 1). Two pairs of diagonal subunits sharing the same conformation are named as Mol I/I’ and Mol II/II’ (Fig. 1b). AKT1 has the same topology as the cyclic nucleotide binding domain (CNBD)-containing channels (Supplementary Fig. 2a), which exhibits a non-domain-swapped subunit arrangement of its voltage sensing domains (VSDs) and pore domains. Each subunit can be divided into four parts: the transmembrane segments comprising S1–S6 and the pore loop; the C-linker which contains the helices A’B’C’D’; and the CNBD (Fig. 1 and Supplementary Fig. 2a). The density map for the N-terminal residues (residues 1–46) and the distal C-terminal region (residues 511–857) is inadequate for model building. At the bottom of the AKT1 map, several densities are only visible in the map at low contour level (threshold 0.015), which may derive from the unresolved ankyrin repeats (ANK) and the KHA domains^16^ (Supplementary Fig. 1b).

Near the C-linkers of Mol I/I’, two short rod-shaped densities are located at the juxta-membrane region. We named them as loops1/1’ (Fig. 1b). In the low contour map, the density of loop1 has contact with Cys331 on C-linker (Supplementary Fig. 2c). The Cα–Cα distance between Cys331 and the interacting residue of loop1 is ~6 Å, which is within the disulfide-bond distance. We next performed mass spectrometry and identified Cys8, at the N-terminal region of AKT1, forms disulfide-bonds with Cys331. Based on this supporting information and the density map, we then built residues 6–14 for loops1/1’.

**Fig. 2:**
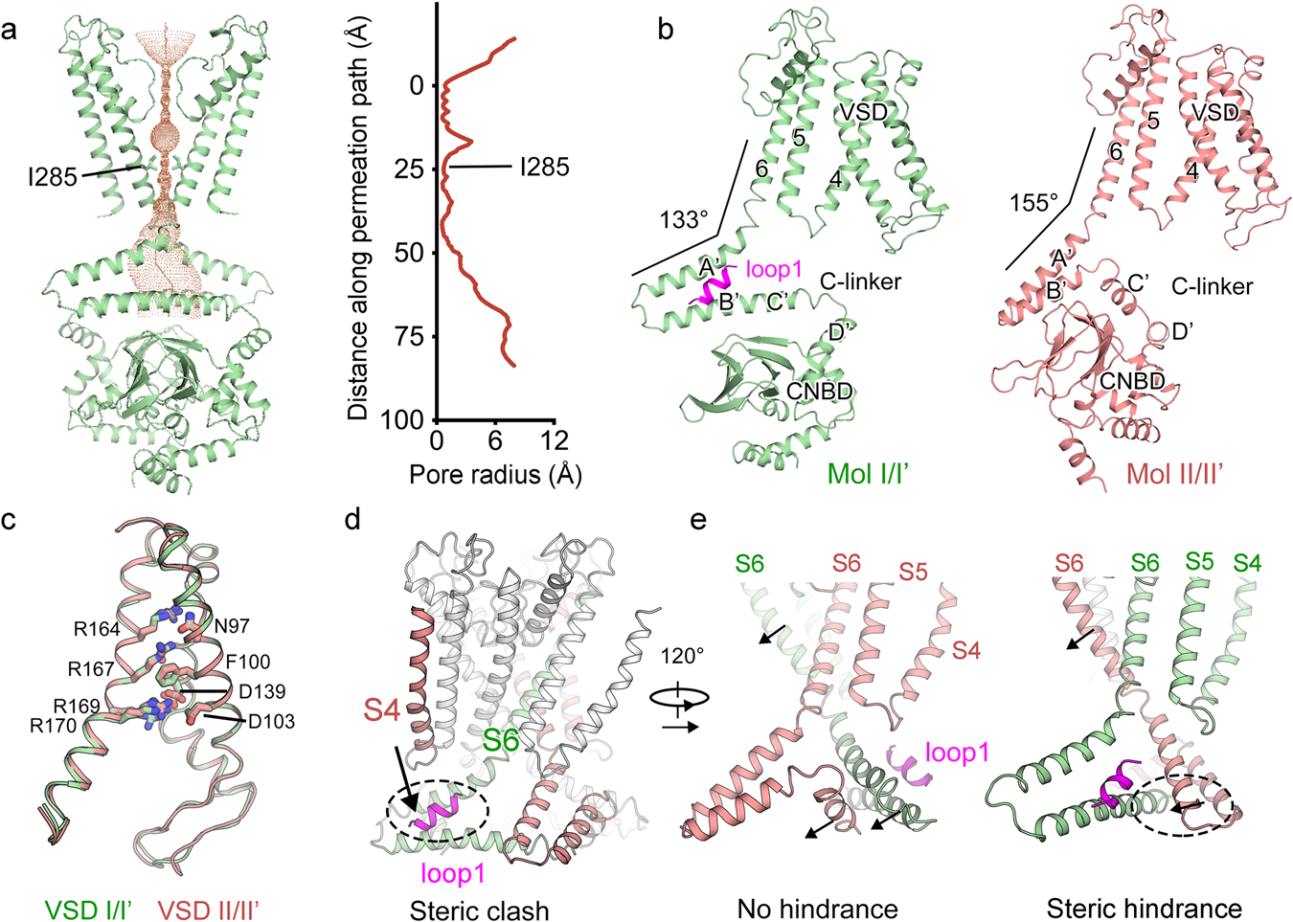
Different conformations of C-linkers lead to the steric hindrance for AKT1 activation. **a** Radius of the pore calculated by the HOLE program^52^. The amino acids restricting the inner gate are shown in sticks. **b** Different conformations of Mol I/I’ (left) and Mol II/II’ (right). **c** VSDs of all four subunits share similar depolarized conformation. **d** Loops1/1’ sterically clashes with the downward movement of S4. **e** The “straight” helix (right) of C-linker sterically affects the movement of the neighboring C-linker, shown in the dashed circle.

The pore domain of AKT1 displays a closed inner gate, with its narrowest constriction formed by I285 (Fig. 2a). This feature functionally corresponds to the expected closed state at 0 mV. Two distinct conformations are adopted by four identical CPDs of AKT1 (Fig. 2b). The voltage sensor of four subunits exhibits depolarized state (Fig. 2c). In Mol I/I’, the helices B’ and C’ of these two C-linkers are pushed into a relatively “straight” helix (Fig. 2b, left panel). The angle between helix A’ and S6 is about 133°. This conformation is different from most C-linker containing channels (Fig. 2b and Supplementary Fig. 3). By contrast, the helices B’ and C’ from C-linkers of Mol II and II’ exhibit a “kinked” conformation. The angle between helix A’ and S6 is about 155° (Fig. 2b and Supplementary Fig. 2). Notably, loops1/1’ sit above the “straight” C-linker, but not the “kinked” C-linker.

For hyperpolarization-activated channels, it has been proposed that a relatively downward movement of S4 is coupled to the reorientation of the C-linker of the adjacent subunit. The C-linkers after these conformational changes will open the S6 gate^17–19^. In our study, loops1/1’ stack against “straight” C-linkers of AKT1 and might affect the downward movement of S4 (Fig. 2d). The two “straight” C-linkers may sterically restrict the conformational change of the neighboring C-linkers, thus inhibiting the opening of the pore (Fig. 2e). These structural findings may underlie the low-activity of AKT1 in the basal state.

In addition, four phospholipids are built surrounding the pore helices of AKT1 (Supplementary Fig. 4). These phospholipids are constitutively associated with AKT1 because no exogenous phospholipids were supplemented during purification. The head groups of these intercalated phospholipids are coordinated by Arg190, Lys193, and Tyr283 on S5 and S6 (Supplementary Fig. 4b, c). Substitutions of these three residues to alanine abrogate AKT1-mediated K^+^ currents (Supplementary Fig. 4d), suggesting that lipids are vital for modulating the activity of AKT1. These observations are reminiscent of the role of phospholipids in KAT1^18,20^. Phospholipids may also regulate the activity of ion channels through protein delipidation and lipidation, illustrated by the mechanosensitive channel MscS^21^.

### Phosphorylation of AKT1 is associated with symmetry rearrangement

To address the activation mechanism triggered by phosphorylation, we sought to determine the structure of phosphorylated AKT1. AKT1 was co-expressed with CIPK23 and CBL1 and purified for cryo-EM study. ATP and sodium orthovanadate (phosphatase inhibitor) were supplemented during AKT1 purification in the attempt to maintain its phosphorylation state (Supplementary Fig. 5a). Phosphorylation sites of AKT1 were firstly identified through mass spectrometry. Compared to AKT1 alone, Ser26 and Ser338 were found to be additionally phosphorylated after co-expression with CIPK23 and CBL1 (Supplementary Fig. 5b).

**Fig. 3:**
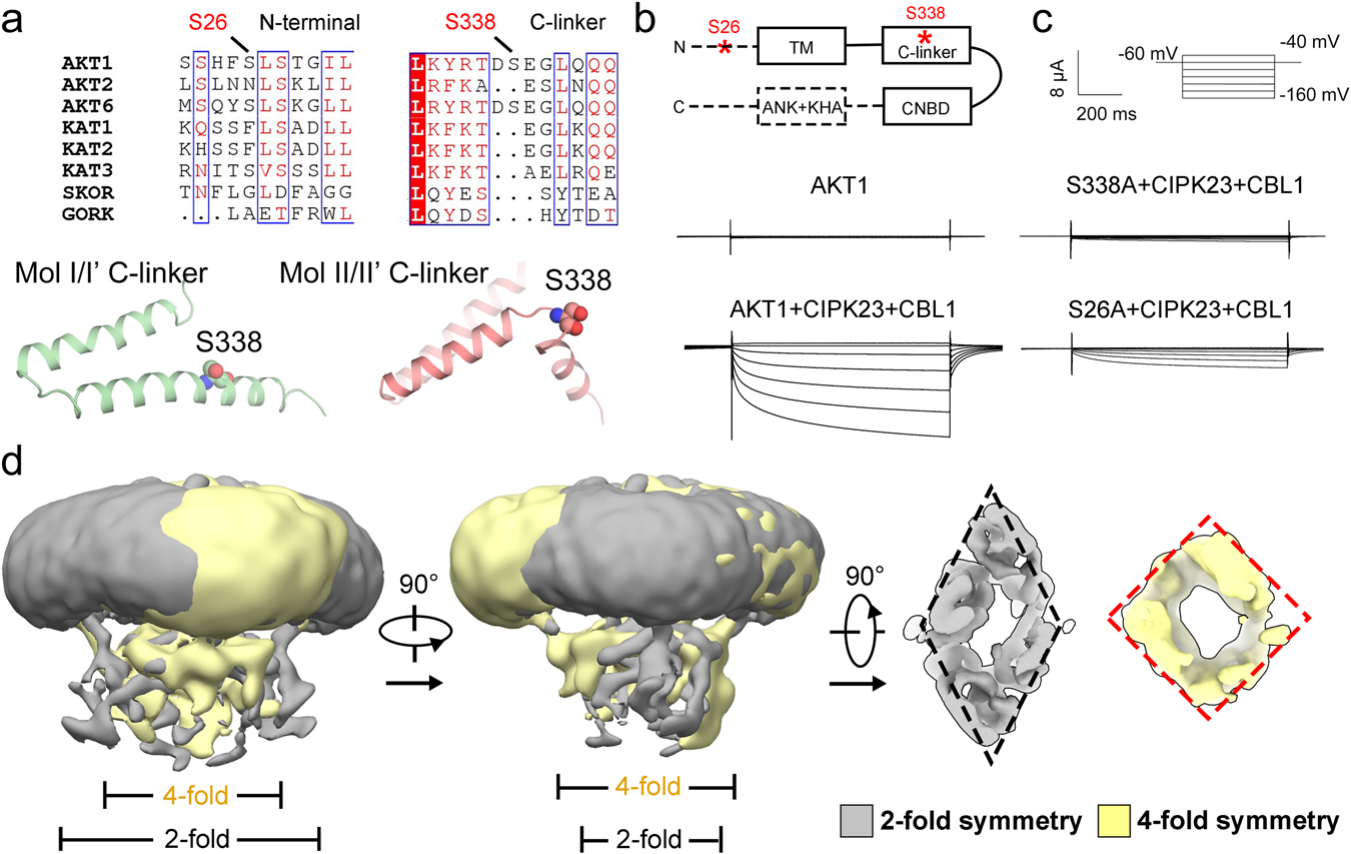
Phosphorylation triggered intra-molecular rearrangement of AKT1. **a** Structural mapping of the identified phosphorylated residues Ser26 and Ser338. A sequence alignment of predominant K^+^ channels in *Arabidopsis* is shown. Ser26 is located at the N-terminus of AKT1. Ser338 is mapped to the kink that differentiates the two conformations of C-linkers. **b** A diagram to show the domain distribution of phosphorylation sites. **c** Whole-cell currents of mutations on phosphorylation sites in the presence of CPIK23 and CBL1. The mutations Ser26Ala and Ser338Ala almost abrogate the K^+^ currents. Three biological repeats were performed with similar results. **d** AKT1 co-expressed with CIPK23 and CBL1 reveals an additional conformation with a 4-fold symmetry at CPD. Source data are provided as a Source Data file.

Ser26 is located at the N-terminus of AKT1, which disallow accurate assignment to the structure. Ser338 locates right on the kink between helices B’ and C’ of the C-linker (Fig. 3a, b). To validate the identified phosphorylation sites, we performed alanine substitution of Ser26 and Ser338 to abolish the phosphorylation. Either Ser26Ala or Ser338Ala severely compromises the currents (Fig. 3c). We next mutated these two residues to aspartic acid to mimic the phosphorylation state. Single mutation of either Ser26Asp or Ser338Asp generates weak K^+^ currents by itself (Supplementary Fig. 5c). Importantly, combined mutation of Ser26Asp and Ser338Asp yield K^+^ currents at −140 mV. The current of double-Asp mutant is relatively weaker than that of wild-type (WT) AKT1 in the presence of CIPK23 and CBL1. This is not surprising because Asp-mimetics cannot completely replace phosphorylation, or more potential phosphorylation sites may exist (Supplementary Fig. 5c).

Cryo-EM analysis was then performed for the phosphorylated AKT1. After 2D classification, several populations of particles assembled in 4-fold symmetry at CPD were found (Supplementary Fig. 5d). 3D reconstruction by these particles yielded a density map in ~4-fold symmetry. The resolution of this map is estimated to only 11 Å (Fig. 3d and Supplementary Fig. 5d), probably because this conformation is highly dynamic and the particle number is relatively small (up to ~6.6%). Nonetheless, these observations enlighten a possibility that the 4-fold symmetry conformation at the CPD may represent a state of AKT1 from silent to active. Supporting this conjecture, another plant K^+^ channel KAT1 with basal activity exhibits 4-fold symmetry^18^. If the symmetry-related conformational change is the underlying mechanism for AKT1 activation, manipulation of the symmetry would alter the channel activity.

### Structure of a constitutively-active variant predominantly assembles in 4-fold symmetry conformation

**Fig. 4:**
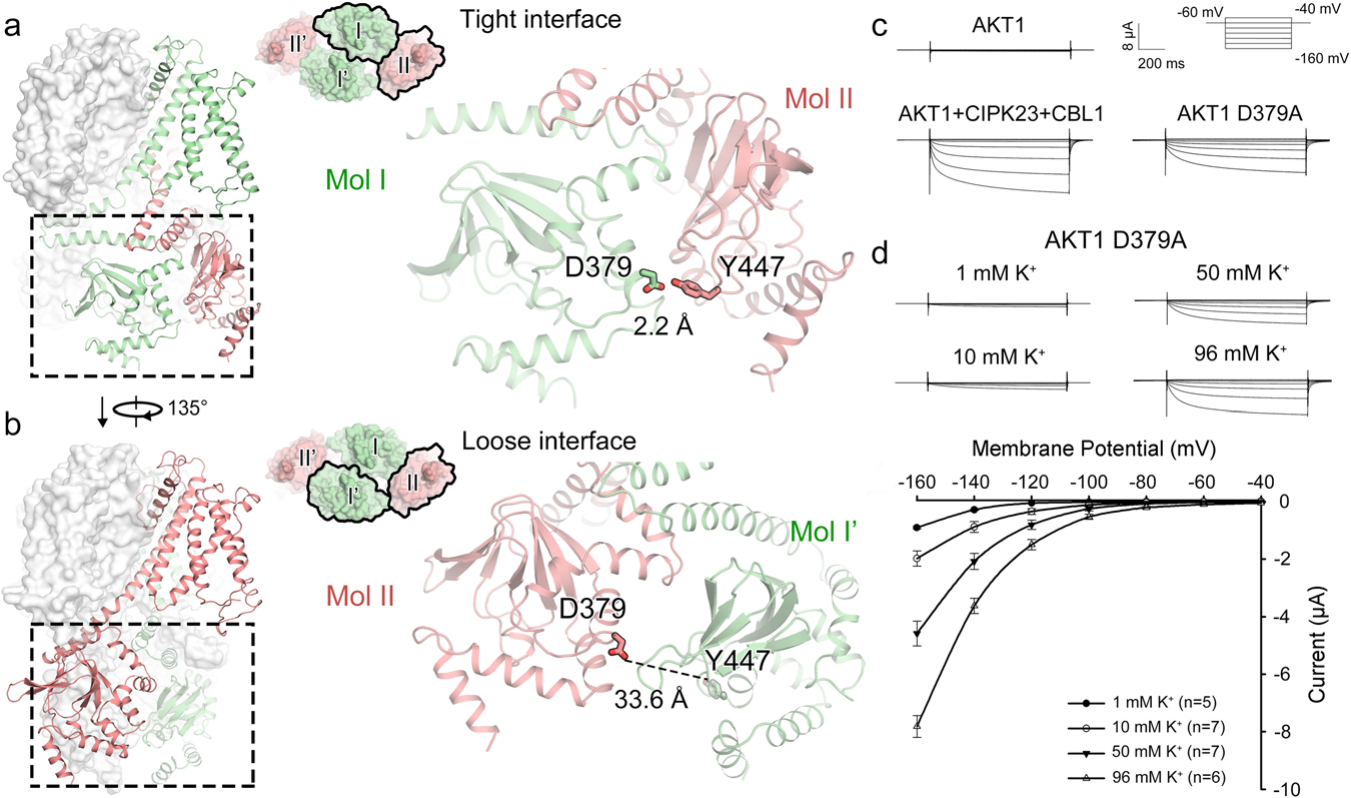
Identification of a constitutively-active mutant by disrupting the dimer interface. **a**, **b** Two different packing interfaces of AKT1. The boxed regions are enlarged in the right panels. Asp379 and Tyr447 are H-bonded at the tight interface. The distance between Asp379 and Tyr447 is 33.6 Å at the loose interface. **c**, **d** Whole-cell currents and I-V curve of a constitutively-active mutant Asp379Ala steady-state currents in various oocytes. Compared to WT AKT1, the point mutation Asp379Ala can mediate K^+^ specific current in the absence of CIPK23 and CBL1. Electrophysiological experiments were repeated using three different batches of oocytes with similar results. Data are presented as means ± SEM. Source data are provided as a Source Data file.

To examine this scenario, we tried to introduce point mutations at the dimer interface of AKT1 CPD. The dimer interfaces of AKT1 CPD can be classified into two types: a tight interface between Mol I and II, and a loose interface between Mol II and I’ (Fig. 4a, b). In the tight interface, Asp379 and Tyr447 of Mol I and Mol II appears to anchor this interface by forming a hydrogen bond (with a distance between the hydroxyl oxygen of Tyr447 and the carboxyl oxygen of Asp379 at 2.2 Å) (Fig. 4a). In contrast, the corresponding distance between Asp379 and Tyr447 in the loose interface is 33.6 Å (Fig. 4b). We individually mutated these two residues to alanine and examined whether they can alter the electrophysiological features of AKT1. Strikingly, Asp379Ala was identified to be a constitutively-active mutant, which could mediate K^+^ currents without CIPK23 and CBL1 (Fig. 4c). The currents of Asp379Ala were external K^+^ dependent, and highly selective for K^+^ (Fig. 4d). The Tyr447Ala mutant also generates currents, but smaller than Asp379Ala (Supplementary Fig. 6). Therefore, the Asp379Ala mutant represents an exceptional candidate to study the activation mechanism of AKT1.

**Fig. 5:**
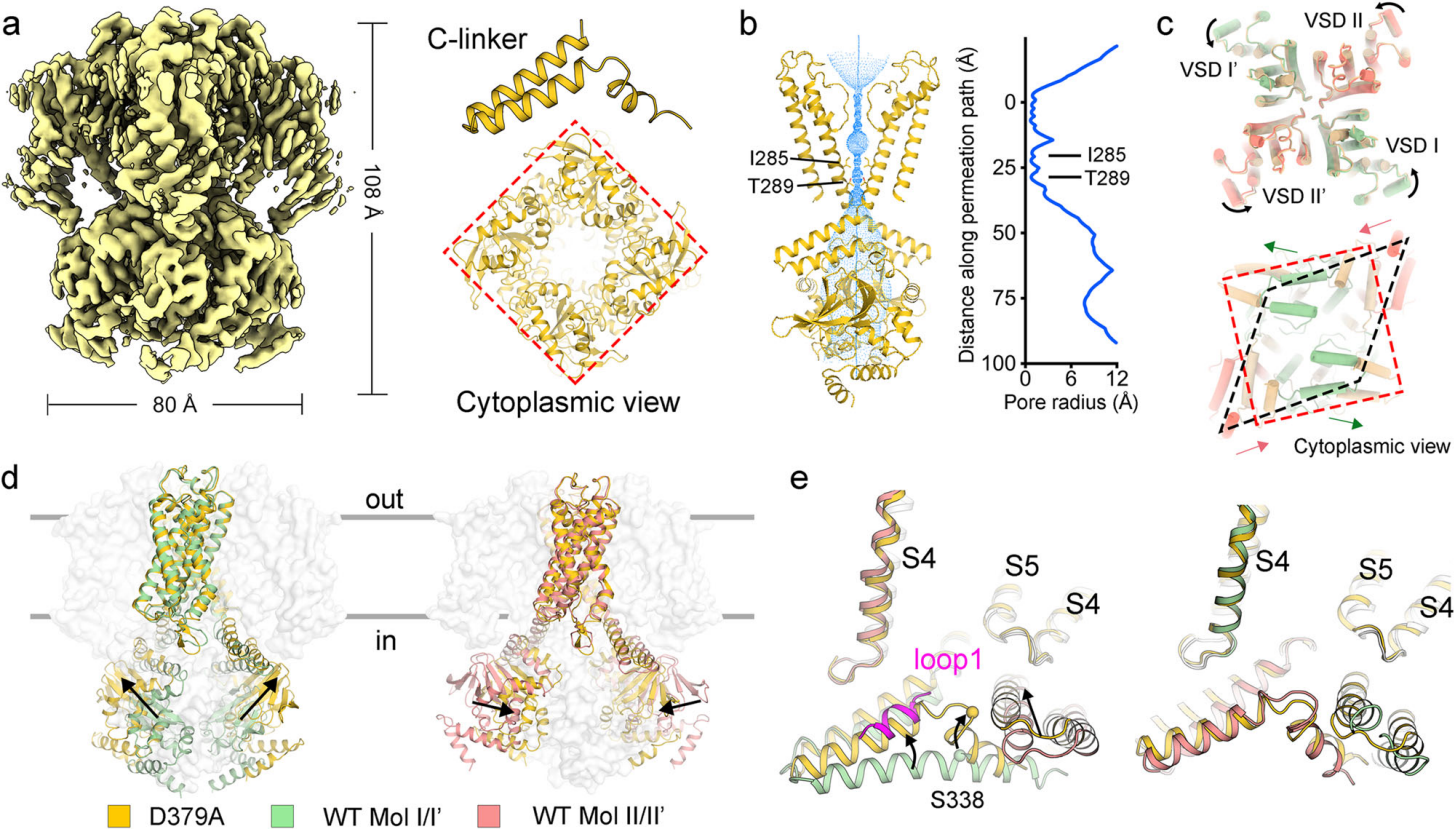
Structure of the constitutively-active AKT1 mutant predominantly adopts a 4-fold symmetry conformation at CPD. **a** Structure of the constitutively-active mutant (Asp379Ala) in 4-fold symmetry at CPD. The C-linkers undergo dramatic conformational change into the canonical kinked conformation. Such conformation eliminates the potential steric hindrance for the adjacent C-linker. **b** Radius of the pore calculated by the HOLE program^52^. The amino acids restricting the inner gate are shown in sticks. **c**, **d** Structural comparison of AKT1 in different symmetric conformations. The CNBDs of Mol I/I’ and Mol II/II’ undergo dramatic conformational change associated with the symmetry rearrangement. **e** Conformational changes of C-linkers and loops1/1’.

The Asp379Ala mutant was then purified to homogeneity for structural study. The 4-fold symmetric CPD was clearly identified from the results of 2D classification (Supplementary Fig. 7a). Several octopus-shaped tails were observed, which were not present in the WT AKT1 (Supplementary Fig. 7a). These tail-like densities might be the unresolved ANKs^22^. The result of 3D classification revealed two separate classes assembled in either 4-fold or 2-fold symmetry. The portion of the particles in 4-fold symmetry substantially increased to ~44%, whereas ~24% particles adopt 2-fold symmetry conformation of the CPD. The particles belonging to the two classes were individually subjected to auto-refinement, yielding two maps at 2.9–3.0 Å (Supplementary Fig. 7 and Supplementary Table 1). In the map with 4-fold symmetric CPD, all four C-linkers exhibit a canonical “kinked” conformation and the width of the CPD is about 80 Å (Fig. 5a). The constriction of inner gate narrows to 0.76 Å, indicating a closed gate (Fig. 5b). These observations imply that the 4-fold symmetry structure of Asp379Ala variant may represent a pre-open state.

Compared to WT AKT1, VSDs of the Asp379Ala variant in 4-fold symmetric model exhibit a slightly counterclockwise rotation (Fig. 5c, upper panel). The CNBDs of Mol I/I’ or Mol II/II’ move reversely with respect to the K^+^ transduction axis (Fig. 5c, lower panel, d). From the side view, the CNBDs of Mol I/I’ tilt up toward the membrane, whereas the CNBDs of Mol II/II’ get close to the K^+^ permeation axis (Fig. 5d). These conformational changes are coupled with the reorientation of C-linkers. The “straight” helix B’C’ of the C-linkers from Mol I/I’ break into two short helices (B’ and C’ helices). The phosphorylation site Ser338 moves upwards about 10.3 Å compared to that on the “straight” C-linker (Fig. 5e). Two C-linkers of the Asp379Ala mutant clash with loops1/1’ from the WT AKT1 (Fig. 5e, left panel). Consistently, the densities of loops1/1’ are invisible in the map containing 4-fold symmetric CPD (Fig. 5a). Such structural changes support the important role of loops1/1’ and C-linkers in modulating the conformation of AKT1.

### AKT1-AtKC1 complex assembles in 2-fold symmetry conformation

**Fig. 6:**
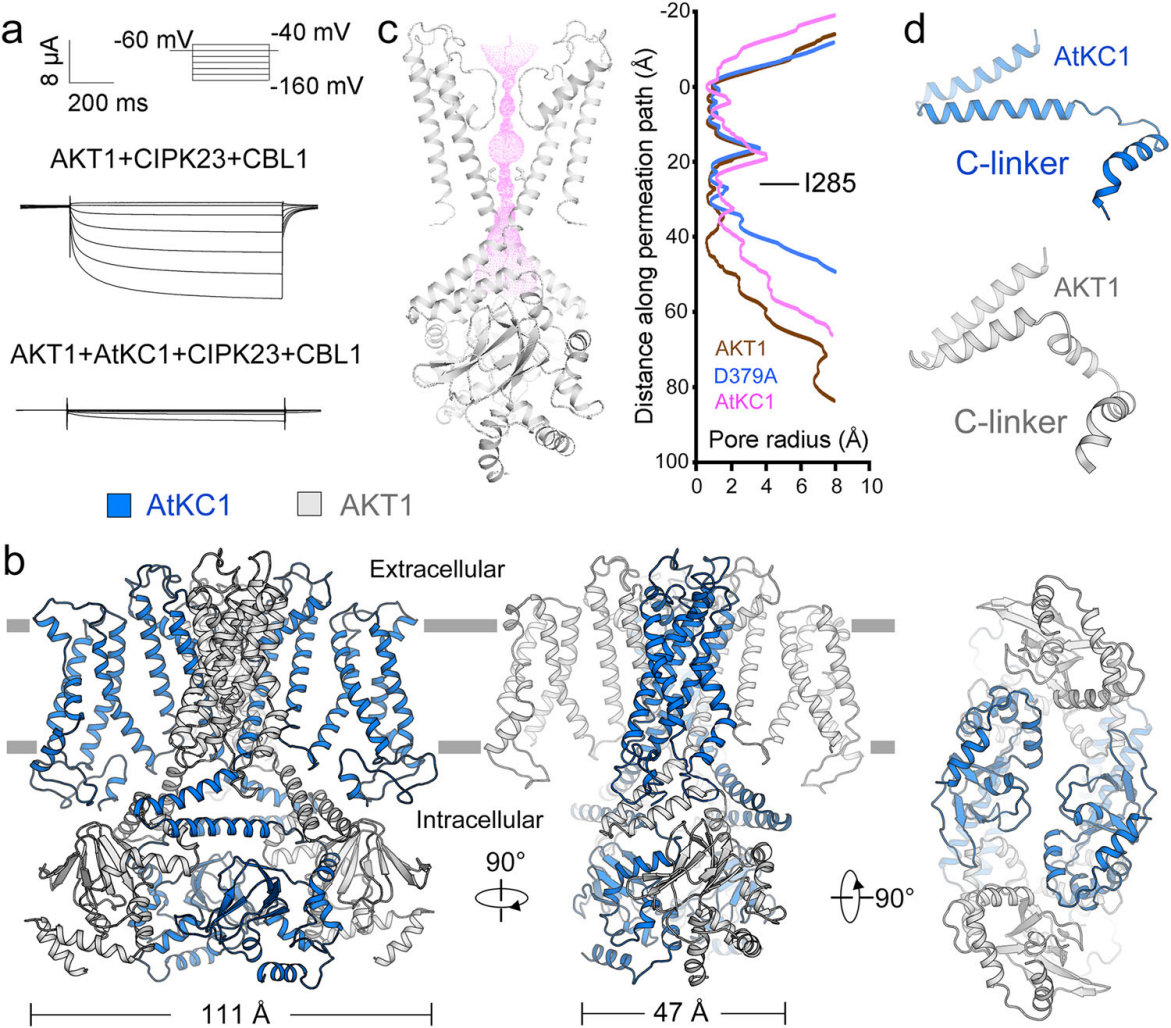
Structure of the AKT1-AtKC1 complex displays 2-fold symmetric assembly. **a** Whole-cell current trace of the AKT1-AtKC1 recorded in *Xenopus* oocytes. AtKC1 negatively regulate the currents in the presence of CIPK23 and CBL1. **b** Structure of the AKT1-AtKC1 in 2-fold symmetry at CPD. **c** Radius of the pore calculated by the HOLE program^52^. The amino acids restricting the inner gate are shown in sticks. **d** The C-linkers of AtKC1 exhibit the “straight” conformation, while the C-linkers of AKT1 displays a kinked conformation. Electrophysiological experiments were repeated using three different batches of oocytes with similar results and one representative image is shown. Source data are provided as a Source Data file.

To further study the conformation-activity relationship of AKT1, we investigated the role of AtKC1 on the regulation of AKT1. The electrophysiological results show that AtKC1 inhibits the channel activity of WT AKT1, the constitutively-active mutant Asp379Ala, and the phosphor-mimetics (Fig. 6a and Supplementary Figs. 8 and 9). Boltzmann analysis of the voltage-dependent activity revealed a half-maximal activation potential (V_1⁄2_) of −101.7 ± 2.0 mV for WT AKT1 in the K^+^ concentration of 96 mM (Supplementary Fig. 10 and Supplementary Table 2). Consistent with previous studies^23,24^, in the presence of AtKC1, the *V*_1⁄2_ shifted by more than −70 mV compared with that of WT AKT1 (Supplementary Table 2). The different voltage thresholds for current activation corresponded to the shift in voltage dependence of the current (Supplementary Fig. 10 and Supplementary Table 2). By contrast, the *V*_1⁄2_ of the constitutively-active mutant of AKT1 shifted about −25 mV upon the presence of AtKC1, suggesting this mutant compromises the inhibitory function of AtKC1. For the phosphor-mimetics including Ser26Asp, Ser338Asp and the double-Asp mutant (Ser26Asp Ser338Asp), the *V*_1⁄2_ values of these mutations all negatively shifted. In the presence of AtKC1, Ser26Asp additionally shifted about −60 mV while the *V*_1⁄2_ of Ser338Asp shifted about −25 mV (Supplementary Fig. 10 and Supplementary Table 2). The *G*_k_ (chord conductance of K^+^) of double-Asp mutant decreased to less than one fifth of WT (Supplementary Fig. 10). Involvement of AtKC1 even reduced the current of double-Asp mutant, which disallows the configuration of the shift of *V*_1⁄2_ and its relative open probability. In addition, all the phosphor-mimetics show steeper curves with lower slope factors (fitted by Boltzmann function) compared to WT AKT1, which indicates similar influence of these mutants on the voltage dependence. Comparison of the *V*_1⁄2_ and the slope factors reveals more important role of Ser338Asp in modulating the voltage dependency. This observation complies with the structural mapping of Ser338Asp to the kink of C-linker that undergoes conformational changes. Similar to the WT AKT1, the voltage dependence of these mutants were changed by AtKC1. To sum up, these results further validate the inhibitory role of AtKC1 toward all the tested AKT1 mutants, though the precise shift values of activation potential are varying for different mutants.

Within expectation, the structure of AKT1-AtKC1 displays a 2-fold symmetric assembly at CPD (Fig. 6b and Supplementary Fig. 11 and Supplementary Table 1). The overall structure of AKT1-AtKC1 is quite similar to WT AKT1, with root-mean-square deviations of 2.78 Å for 1753 aligned Cα atoms. The inner gate of the hetero-channel is also in closed state (Fig. 6c). Markedly, the C-linkers of AtKC1 resemble the “straight” C-linkers of AKT1 Mol I/I’, while the C-linkers of AKT1 exhibit the canonical “kinked” conformation as Mol II/II’ (Fig. 6d). The “straight” C-linkers of AtKC1 may restrict the movement of adjacent C-linkers of AKT1. This structural observation mechanistically explains how AtKC1 exerted its inhibitory effect on AKT1. Different from WT AKT1, no extra densities that resemble loop1/1’ of AKT1 have been observed near the “straight” C-linker of AtKC1. This indicates the C-linkers of AtKC1 preferentially adopt a “straight” conformation, without the help of loop1/1’-like elements.

## Discussion

The structures of AKT1 reported here reflect two different intra-molecular organizations. In the silent state, AKT1 exhibits 2-fold symmetric assembly of the CPD with two “straight” C-linkers and N-terminal loops1/1’. This represents an autoinhibited conformation. In the AKT1-AtKC1 complex, the CPD is assembled in 2-fold symmetry, with the C-linkers of AtKC1 exhibit a “straight” conformation as well. By contrast, the constitutively-active mutant Asp379Ala mainly displays 4-fold symmetric conformation of the CPD. Compared to WT AKT1, the original “straight” C-linkers change to the “kinked” conformation, accompanied with the leaving of loops1/1’ (Fig. 5a, e). Taken together, the “straight” C-linkers and loops1/1’ are two featured structural elements that act crucial roles in the formation of the 2-fold symmetric assembled CPD. During preparation of this manuscript, another group independently described the 2-fold symmetric structure of WT AKT1. Consistent with our study, they also observed the “straight” C-linkers. The similar densities corresponding to the loops1/1’ were described as N-terminal helices in their work^25^ (Supplementary Fig. 12a).

We noticed that different conformations of C-linkers are closely associated with the presence or absence of loops1/1’ in AKT1. We reason that loops1/1’ may help to maintain the “straight” conformation of C-linkers, whereas departure of loops1/1’ would release the space to accommodate the “kinked” C-linkers. The tilt-up C-linkers then drag the CPD to form a 4-fold symmetric assembly. This series of coordinated rearrangements may eventually lead to the activation of AKT1. If the leaving of loops1/1’ is required for the activation of AKT1, then weaken the interaction between loops1/1’ and C-linkers may generate K^+^ currents and lead to a 4-fold symmetric CPD. To examine such scenario, we deleted the N-terminal 15 residues (AKT1_16-857_) and introduced a point mutation Cys8Ala to break the disulfide bonds between loops1/1’ and C-linkers. As expected, both variants alone can generate weak currents, and the presence of CIPK23 and CBL1 enhance the K^+^ transduction (Supplementary Fig. 12b). Notably, cryo-EM analysis of purified AKT1_16-857_ reveals particles with 4-fold symmetric CPD during 2D classification (Supplementary Fig. 12c). These results clearly demonstrate the regulatory effect of the N-terminal loops1/1’. However, neither deletion of loops1/1’ nor Cys8Ala was able to completely replace the role of phosphorylation. Possibly because phosphorylation would trigger more dramatic structural rearrangement, including pushing the loops1/1’ away from the C-linkers and breaking the “straight” C-linkers to “kinked” ones. It is noteworthy that the identified phosphorylation sites, Ser26 and Ser338, are located around the loops1/1’ and C-linkers respectively, where dramatic conformational change occurs upon channel activation (Supplementary Fig. 12d).

Such model may also provide clues to mechanistically explain the activation difference of AKT1 and AKT6 (also SPIK or AT2G25600 in Uniprot and TAIR database). Among several fundamental K^+^ channels in *Arabidopsis*, AKT6 shares high sequence identity with AKT1 (Fig. 3a and Supplementary Fig. 13). AKT6 has been reported to exhibit basal activity^26^. Preliminary cryo-EM analysis of AKT6 reveals 4-fold symmetry features at CPD (Supplementary Fig. 13). Although Ser26 and Ser338 are conserved between AKT1 and AKT6, AKT6 lacks the N-terminal cysteine (Cys8 in AKT1), which may ultimately contribute to their different activation mechanism.

Phosphorylation has been widely reported to modulate the function of ion channels or transporters in plants. For example, phosphorylation modulates the activity of SLAH/SLAC anion channels through stoichiometry change^27,28^. And phosphorylation by CIPK23 and CBL1 may activate HAK5 by releasing the autoinhibitory domain^29^. For AKT1, the phosphorylation level, but not the expression level was thought to mediate the response to K^+^ alteration^5^. Such information suggests that AKT1 are not activated simultaneously, and the fluctuation of phosphorylation level of AKT1 is required for the balance of K^+^ concentration in cells. The CIPK family members phosphorylate only small portions of target proteins^5,30–32^. This fact may explain why only a small portion of phosphorylated AKT1 protein sample was found to display 4-fold symmetric conformation at CPD.

To our knowledge, AKT1 is a characteristic ion channel that the activity can be modulated with a 2-fold to 4-fold symmetry switch by itself or an α-subunit. Although the 2-fold symmetric assemblies of the CPD were also reported in a zebrafish ELK channel and a crystal structure of the EAG domain-CNBHD complex of mouse EAG1, the intact structures of these two channels in multiple conformations await further structural and functional examinations^33,34^. Why four identical subunits can be assembled in both 2-fold and 4-fold symmetry at the cytoplasmic side? AKT1 constitutes the primary K^+^ channel in root cells, and is regarded as a K^+^ sensor^35^. Uptake of K^+^ participates in anti-salt stress process as well^36,37^. Both roles of AKT1, as a sensor and an anti-stress executant, require strict regulation of the channel activity. AKT1 mostly adopts a 2-fold symmetric conformation, as low-energy favored state, in silent state. Upon stimuli of environmental stress or endogenous lack of K^+^, the conformation of AKT1 can be induced to 4-fold symmetric conformation, which exhibit high performance for K^+^ conduction. The observation of 2-fold symmetric conformation of AKT1-AtKC1 complex also provides further clues for interpreting such regulatory mechanism.

We keenly recognize that the above hypothesis is speculative and limited, because the activity of AKT1 is subject to additional layers of regulation. For example, the ANK and KHA domains are reported to regulate the channel activity of AKT1^7,16^. However, these domains could not be well-assigned in the structure. We noticed the variation of the tail-like densities in the maps between the WT and the variant at low contour level (Supplementary Fig. 14). In the map of WT AKT1, the probable densities of ANKs displays 2-fold symmetry features (Supplementary Fig. 14a). By sharp contrast, the ANK densities in the 4-fold symmetric exhibit a “down” conformation (Supplementary Fig. 14b). The crystal structure of the ANKs can be docked into the density^22^ (Supplementary Fig. 14). CIPK23 has been reported to bind ANKs^7,22^. Thus, different conformations of ANKs and the binding of CIPK23 may be involved in the activity regulation.

In this study, we report a regulatory mechanism of AKT1 activity through conformational changes associated with symmetric rearrangement. Because the sequences of AKT1 are conserved in its orthologue from maize and rice^38–41^, we anticipate that our work may provide a framework for understanding these channels and hold the promise to optimize K^+^-deficiency resistant phenotype of corps. These results may also gain insights into functional studies and engineering of other ion channels.

## Methods

### Purification of *Arabidopsis* potassium channel AKT1

The coding sequence of *Arabidopsis thaliana* AKT1 was subcloned into the pFastBac1 (Invitrogen) vector with a N-terminal Flag tag. The recombinant AKT1 was expressed using the Bac-to-bac system (Invitrogen). Briefly, bacmids were generated in DH10Bac competent cells, and the resulting baculoviruses productions were generated and amplified in Sf9 insect cells (Invitrogen). AKT1 was overexpressed in Sf9 insect cells grown in the Serum-Free Medium (Gibco). Sixty hours after P3 virus infection, transfected cells were collected and homogenized in the buffer containing 25 mM Tris-HCl, pH 7.4, 150 mM KCl supplemented with protease inhibitor cocktails containing 1 mM phenylmethylsulfonyl fluoride (PMSF), 1.3 μg/ml aprotinin, 0.7 μg/ml pepstatin and 5 μg/ml leupeptin. After brief sonication, the suspension was supplemented with n-dodecyl-β-D-maltopyranoside (DDM, Anatrace) to a final concentration of 2% (w/v) and cholesteryl hemisuccinate Tris salt (CHS, Anatrace) to 0.2% (w/v). After incubation at 4 °C for 2 h, the mixture was centrifuged at 150,000 × *g* for 30 min, and the supernatant was applied to 1 ml anti-Flag M2 affinity gel (Sigma) by incubating at 4 °C. The affinity gel was rinsed with buffer containing 25 mM Tris-HCl, pH 7.4, 150 mM KCl, 0.1% DDM and 0.01% CHS for three times. Then the target protein was eluted with wash buffer plus 200 μg/ml flag peptide. Eluted protein was concentrated with a 50-kDa cut-off Centricon (Millipore) and further purified by Superose-6 column (GE Healthcare) in the buffer containing 25 mM Tris-HCl, pH 7.4, 150 mM KCl, and 0.1% digitonin. The peak fractions were collected for cryo-EM analysis. The AKT1 variant was generated with a standard PCR-based strategy and subcloned into the pFastBac1 (Invitrogen) vector with a N-terminal Flag tag. The recombinant AKT1-D379A was expressed using the Bac-to-bac system (Invitrogen). Purification of AKT1-D379A follows the same protocol as that of AKT1.

To obtain the phosphorylated AKT1, CIPK23 and CBL1 were co-expressed with AKT1 in HEK293 cells, which may be more suitable for phosphorylation. We supplemented 1 mM ATP during the whole purification process to maintain the phosphorylation states of AKT1. At the same time, 1 mM sodium orthovanada was added to inhibit potential dephosphorylation by endogenous phosphatases. The harvested cells were resuspended in 25 mM Tris-HCl, pH 7.4, 150 mM KCl, 1 mM sodium orthovanada, 1 mM PMSF, 1.3 μg/ml aprotinin, 0.7 μg/ml pepstatin and 5 μg/ml leupeptin. In total, 2% DDM, 0.2% CHS, 0.5 mM CaCl_2_, 5 mM MnCl_2_, 1 mM ATP and 1 mM sodium orthovanada were supplemented for whole-cell extract^5^. The supernatant after ultracentrifugation was applied to affinity purification using anti-Flag M2 gel. The elution was subjected to gel filtration in the buffer containing 0.08% digitonin, 0.5 mM CaCl_2_, 5 mM MnCl_2_, 1 mM ATP, 1 mM sodium orthovanada. The peak fractions were applied to cryo-EM sample preparation. AKT1 alone was also expressed in HEK293F cells. Both AKT1 in the absence or presence of CIPK23 and CBL1 were transferred to mass spectrometry for phosphorylation identification.

### Purification of *Arabidopsis* AKT1-AtKC1 complex

To obtain the AKT1-AtKC1 complex, AKT1 and AtKC1 were co-expressed in HEK293 cells. The full-length *Arabidopsis thaliana* AKT1 was subcloned in pCAG vector with an N-terminal Flag, while the full-length AtKC1 was subcloned in pCAG vector with an N-Twin Strep. HEK293F cells were transformed for protein expression. Cells were collected 60 h after transformation, homogenized in the buffer containing 25 mM Tris-HCl, pH 7.4, 150 mM KCl supplemented with protease inhibitor cocktails containing 1 mM PMSF, 1.3 μg/ml aprotinin, 0.7 μg/ml pepstatin and 5 μg/ml leupeptin. In total, 1% DDM, 0.1% CHS were supplemented for whole-cell extract^5^. The supernatant after ultracentrifugation was applied to affinity purification using anti-Flag M2 gel. The elution was adjusted to pH 8.0 by 1 M Tris-HCl, pH 8.0, then applied to 1 ml Streptactin Beads 4FF (Smart lifesciences) by incubating at 4 °C. The affinity gel was rinsed with buffer containing 25 mM Tris-HCl, pH 8.0, 150 mM KCl, 0.1% DDM and 0.01% CHS for three times. Then the target protein was eluted with wash buffer plus 2.5 mM desthiobiotin. Eluted protein was concentrated with a 50-kDa cut-off Centricon (Millipore) and further purified by Superose-6 column (GE Healthcare) in the buffer containing 25 mM Tris-HCl, pH 7.4, 150 mM KCl, and 0.06% digitonin. The peak fractions were collected for cryo-EM analysis.

### Preparation of the cryo-EM samples

Aliquots of 4 μl AKT1 was dropped onto glow-discharged holey carbon grids (Quantifoil Au R1.2/1.3, 300 mesh). The grids were blotted for 3 s at 8 °C under 100% humidity and flash-frozen in liquid ethane using Vitrobot Mark IV (FEI). The sample was imaged on an FEI 300 kV Titan Krios transmission electron microscope equipped with a Cs corrector and Gatan GIF Quantum energy filter (slit width 20 eV), recorded by a Gatan K2 Summit detector with a nominal magnification of ×81,000. A series of defocus values from −1.5 to −1.8 µm was used during data collection. Each image was dose-fractionated to 32 frames with a total electron dose of ~50 e^−^ Å^−2^ and a total exposure time of 5.6 s. AutoEMation II (developed by J. Lei)^42^ was used for fully automated data collection. All stacks were motion-corrected using MotionCor2 with a binning factor of 2^43^, resulting in a pixel size of 1.0825 Å. The defocus values were estimated using Gctf^44^ and dose weighing was performed concurrently^45^. The images of AKT1 co-expressed with CIPK23 and CBL1 was recorded by Gatan K2 Summit detector with a nominal magnification of ×29,000. After motion-correction with a binning factor of 2, resulting in a pixel size of 0.97 Å. The images of AKT1-AtKC1 were recorded by Falcon4 detector with a nominal magnification of ×96,000. After motion-correction with a binning factor of 2, resulting in a pixel size of 0.86 Å. EPU was used for automated data collection.

### Cryo-EM data processing

In total 349,544 particles were auto-picked from these 1017 movie stacks using Gautomatch (developed by Kai Zhang, http://www.mrc-lmb.cam.ac.uk/kzhang/Gautomatch) (Supplementary Fig. 1). After 2D classification, 222,790 particles were selected. The 222,790 particles were subjected to 50 iterations of global angular search 3D classification. Each of the 50 iterations has one class and a step size of 7.5°. For each of the last five iterations (iteration 46–50) of the global search, the local angular search 3D classification was executed with a class number of 4, a step size of 3.75°, and a local search range of 15°. A total of 181,714 particles were selected from the local angular searching 3D classification after removing the redundant particles and particles from bad classes (Supplementary Fig. 1). The selected particles were subjected to 3D autorefinement, yielding a density map with average resolution at 3.4 Å in C1 symmetry and C2 symmetry on the basis of the Fourier shell correlation (FSC) 0.143 criterion. The FSC curves were corrected for the effects of a soft mask using high-resolution noise substitution. Local resolution variations were estimated using RELION-3.1^46^.

To analyze the structure of AKT1 in the presence of CIPK23 and CBL1, 1,224,092 particles were auto-picked from these 5711 movie stacks. In total, 827,944 particles were selected after 2D classification. We randomly selected several classes from the side view and the classes that exhibit 4-fold symmetry for 3D classification. In total 54,586 particles were subjected to 3D classification for the 4-fold symmetry assembled model. However, the particles of phosphorylated AKT1 occupies a small portion due to the highly dynamics of phosphorylation-dephosphorylation process. Nonetheless, we can clearly discriminate the two conformations of AKT1 (Supplementary Fig. 5).

For the constitutively-active variant, 1,737,511 particles were auto-picked from these 5433 movie stacks (Supplementary Fig. 7). In total, 758,925 particles were selected after 2D classification. The 758,925 particles were subjected 3D classification. Two classes in 4-fold and 2-fold symmetry are identified simultaneously. The selected particles from the two classes were subjected to auto-refinement, yielding two density maps with average resolution at 2.9–3.0 Å.

For the cryo-EM analysis of AKT1-AtKC1, 6576 movie stacks have been collected and processed following the tutorial of Cryosparc^47^ (Supplementary Fig. 9). In total 104,142 particles were used to generate the density map with average resolution at 3.3 Å.

### Model building and structure refinement

The model of AKT1 was built de novo from a poly-Ala model. Sequence assignment was guided by bulky residues such as Tyr, Phe and Trp. The structure was then refined in real space using PHENIX with secondary structure and geometry restraints^48^. The atomic model was manually improved using COOT^49^. Four lipid densities at the interfaces of two subunits were built as phosphatidylethanolamine. Several EM density lobes resemble phospholipids; but the quality of these densities was insufficient for assignment of the phospholipids. This final atomic model was refined in real space using PHENIX. The final atomic model was evaluated using MolProbity^50^. The model of AKT1 was used as a template to build the model of the variant.

### In vitro transcription and expression in *Xenopus* oocytes

The coding sequences of AKT1, CIPK23, and CBL1 were cloned into pGEM-HE vector. All AKT1 variants mentioned in this study were cloned into pGEM-HE vector. The cRNAs were transcribed in vitro using the mMESSAGE mMACHINE™ T7 Transcription Kit (Invitrogen). Oocytes were isolated from *X. laevis* and injected with cRNAs. The oocytes were injected with nuclease-free water (25 nl as control), AKT1 cRNA (8 ng in 25 nl), AKT1 and CIPK23 cRNA mixture (8:4 ng in 25 nl), AKT1 and CBL1 cRNA mixture (8:4 ng in 25 nl), AKT1 and AtKC1 cRNA mixture (8:8 ng in 25 nl), the cRNA mixture of AKT1, CIPK23, and CBL1 (8:4:4 ng in 25 nl), the cRNA mixture of AKT1, AtKC1, CIPK23, and CBL1 (8:8:4:4 ng in 25 nl), respectively. Before used in voltage-clamp recordings, the injected oocytes were incubated in ND96 solution containing 96 mM NaCl, 2.0 mM KCl, 1.8 mM CaCl_2_, 1.0 mM MgCl_2_·6 H_2_O, and 5 mM HEPES-NaOH, pH 7.5, supplemented with 0.1 mg/ml gentamycin at 17 °C for 40 h before electrophysiological recording.

### Two-electrode voltage-clamp recording from *Xenopus* oocytes

A two-electrode voltage-clamp technique was applied using a GeneClamp 500B amplifier (Axon Instruments) at room temperature. The microelectrodes were filled with 3 M KCl. The bath solution contained 96 mM KCl, 1.8 mM MgCl_2_·6 H_2_O, 1.8 mM CaCl_2_, and 10 mM HEPES-NaOH, pH 7.2. Voltage steps were applied from +40 to −180 mV in −20 mV or −10 mV decrements during 1.0 s, from a holding potential of −60 mV. Each step begins with 0.21 s and ends with 0.19 s at the resting potential of the oocyte membrane in the tested bath solution. Whole-cell currents were filtered at 1 kHz and digitized through a Digidata 1322A AC/DC converter using Clampex 9.0 software (Axon Instruments). The relative open probability of AKT1 is parameterized with the Boltzmann function, *G*/*G*_max_ (relative open probability) = 1/(1 + exp((*V*_m_ − *V*_1/2_)/*S*)). *G* (chord conductance) was calculated as *G* = *I*/(*V*_m_ − *E*_K_), where *I* is the steady-state current at voltage *V*_m_, *E*_K_ is the reversal voltage, *V*_1/2_ is the half-maximal activation potential and *G*_max_ is the maximal conductance. The steepness of the voltage-dependence is either described by the so-called “slope factor”, *S* (in mV), or the “apparent gating valence” *z*_g_ (dimensionless). These two quantities are inversely related by *S* = *RT*/*z*_g_*F*, and the factor *RT*/*F* amounts to ~24 mV at room temperature^51^. The data are presented as means ± SE.

### Sample preparation and mass spectrometry

SDS-PAGE bands were excised for in-gel digestion, and peptides derived from the extracted proteins were subsequently analyzed by mass spectrometry. Briefly, WT AKT1 bands were subjected to in-gel digestion using sequencing-grade modified trypsin in 50 mM ammonium bicarbonate at 37 °C overnight. The resulting peptides were extracted twice with 1% trifluoroacetic acid in 50% acetonitrile aqueous solution for 30 min. The peptide extracts were then centrifuged in a SpeedVac to reduce the volume.

For LC-MS/MS analysis, peptides were separated by a 120 min gradient elution at a flow rate 0.300 μl/min with a Thermo-Dionex Ultimate 3000 HPLC system, which was directly interfaced with the Thermo Orbitrap Fusion mass spectrometer. The analytical column was a homemade fused silica capillary column (75 μm ID, 150 mm length; Upchurch, Oak Harbor, WA) packed with C-18 resin (300 A, 5 μm; Varian, Lexington, MA). Mobile phase A consists of 0.1% formic acid, and mobile phase B consists of 100% acetonitrile and 0.1% formic acid. The Orbitrap Fusion mass spectrometer was operated in the data-dependent acquisition mode using Xcalibur (version 3.0) software. Each MS1 full-scan was performed by the Orbitrap (350–1550 *m*/*z*; AGC target, 4e^5^; maximum injection time, 50 ms; 120,000 resolution) followed by 3 MS/MS scans (AGC, 5e^4^; maximum injection time, 45 ms; 30,000 resolution) with fragmentation in the Ion Routing Multipole (normalized collision energy (HCD), 30%).

For identification of disulfide bond, the MS/MS spectra from each LC-MS/MS run were searched against the user-curated database (*Arabidopsis thaliana* AKT1 protein sequence downloaded from Uniprot, Q38998) using pLink (version 1.9). The linker was set disulfide bond (HCD-SS). Peptides were required to possess 20 ppm precursor accuracy and 0.02 Da fragment ion accuracy. Peptides with a length between 6 and 60 were selected for searching, with a minimum mass of 600 Da and a maximum mass of 6000 Da per chain. Variable methionine oxidation (+15.994915 Da) and static cysteine carbamidomethylation (+57.021 Da) were included as modifications. Trypsin was specified for digestion, and a maximum of two missed cleavage sites was allowed for each peptide. Peptide fragmentation data were reported at 1% false discovery rate in Scafflod 4.5. The identified peptides containing disulfide-bonds were manually validated.

For identification of phosphorylation sites, the MS/MS spectra from each LC-MS/MS run were searched against the user-curated database (*Arabidopsis thaliana* AKT1 protein sequence downloaded from Uniprot, Q38998) using Sequest HT node in Proteome Discoverer (version 1.4). Peptides were required to possess 20 ppm precursor accuracy and 0.02 Da fragment ion accuracy. The following modifications were included: variable oxidation of methionine (+15.994915 Da) and phosphorylation of serine, threonine, and tyrosine (+79.966 Da), as well as static carbamidomethylation of cysteine (+57.021 Da). Trypsin was specified for digestion, and a maximum of two missed cleavage sites was allowed for each peptide. The phosphopeptides were verified using the phosphoRS 3.1 node in Proteome Discoverer software, with a cutoff of 0.75. Peptide fragmentation data were reported at 1% false discovery rate. All MS/MS spectra corresponding to phosphopeptides were manually examined.

### Ethics declarations

Animal studies were conducted in accordance with the ethical guidelines of Ministry of Agriculture (Beijing, China). The animal experiments conformed to the guidelines and regulatory standards of the Institutional Animal Care and Use Committee of China Agricultural University, no. AW20902202-3-1.

### Statistics and reproducibility

The experiments in Figs. 1a, 3c, 4c, d, 6a and Supplementary Figs. 4d, 5c, 6, 8–10, 12b were repeated at least three times using three different batches of oocytes. Similar results were obtained.

### Reporting summary

Further information on research design is available in the Nature Research Reporting Summary linked to this article.

## Supplementary information

Supplementary Information

Reporting Summary

## Data Availability

The data that support this study are available from the corresponding authors upon reasonable request. The cryo-EM maps have been deposited in the Electron Microscopy Data Bank (EMDB) under the accession code EMD-32769 (AKT1 WT), EMD-31532 (AKT1 Asp379Ala, constitutively-active mutant), and EMD-33467 (AKT1-AtKC1 complex). The atomic coordinates for the corresponding models have been deposited in the Protein Data Bank (PDB) under the accession code 7WSW (AKT1 WT), 7FCV (AKT1 Asp379Ala, constitutively-active mutant), and 7XUF (AKT1-AtKC1 complex). Source data are provided with this paper.

## Source data

Source Data
