# Supplementary material for: Structural basis for the activity regulation of a potassium channel AKT1 from Arabidopsis
Source: Nat Commun. 2022 Sep 27;13:5682. doi: 10.1038/s41467-022-33420-8 (PMC9515098; doi:10.1038/s41467-022-33420-8)
Supplement: Supplementary file 1 — Supplementary Information [file 41467_2022_33420_MOESM1_ESM.pdf]

Supplementary Information for

Structural basis for the activity regulation of a potassium channel AKT1 from Arabidopsis

Yaming Lu[1*], Miao Yu[2*], Yutian Jia[1*], Fan Yang[1], Yanming Zhang[1], Xia Xu[1], Xiaomin Li[3], Fan Yang[3], Jianlin Lei[3], Yi Wang[2,4], and Guanghui Yang[1,4]

[1]State Key Laboratory for Agrobiotechnology, Frontiers Science Center for Molecular Design Breeding, College of Biological Sciences, China Agricultural University, Beijing 100193, China

[2]State Key Laboratory of Plant Physiology and Biochemistry, College of Biological Sciences, China Agricultural University, Beijing 100193, China

[3]Technology Center for Protein Sciences, Ministry of Education Key Laboratory of Protein Sciences, School of Life Sciences, Tsinghua University, Beijing 100084, China.

[4]Correspondence to Yi Wang (yiwang@cau.edu.cn) and Guanghui Yang (guanghuiyang@cau.edu.cn).

[*]These authors contributed equally to this work.

This PDF file includes:

Supplementary Figs. 1-14
Supplementary Table 1-3

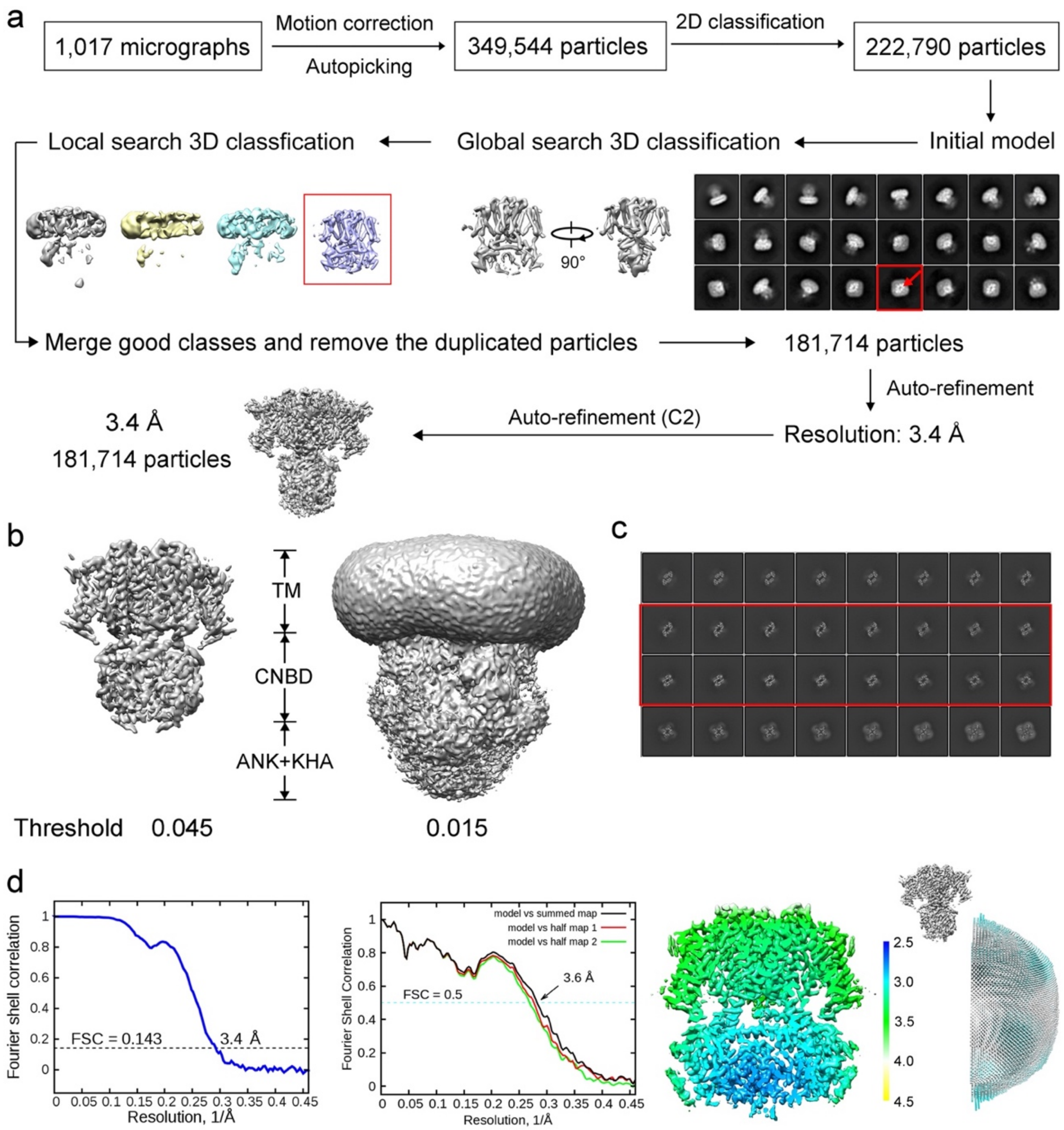

Supplementary Fig. 1 | Cryo-EM analysis of hyperpolarization-activated potassium channel AKT1. **a,** A flowchart description of EM data processing for AKT1. The average resolution for the final reconstruction of AKT1 is estimated to be 3.4 Å. Representative micrograph and 2D class averages of AKT1. Details are described in the Methods. **b,** 3D classification result of AKT1 presented with different threshold in Chimera. **c,** Cut-view of the EM map reveals 2-fold symmetric assembly of AKT1 in

the cytoplasmic side (red box) but 4-fold symmetry in the transmembrane region. **d,** Cryo-EM analysis of AKT1. The overall resolution of the reconstruction for AKT1 is estimated to be 3.4 Å on the basis of the FSC 0.143 value (left). The FSC curves of the refined model versus the map that it is refined against (black); of the model refined in the first of the two independent maps used for the FSC calculation versus that same map (red); and of the model refined in the first of the two independent maps versus the second independent map (green). The small difference between the red and green curves indicates that the refinement did not suffer from overfitting (middle). Local resolution distribution of the final reconstruction for AKT1 as estimated by RELION-3.1[6]. Angular distribution of the particles used for reconstruction of AKT1. Each cylinder represents one view and the height of the cylinder is proportional to the number of particles for that view (right).

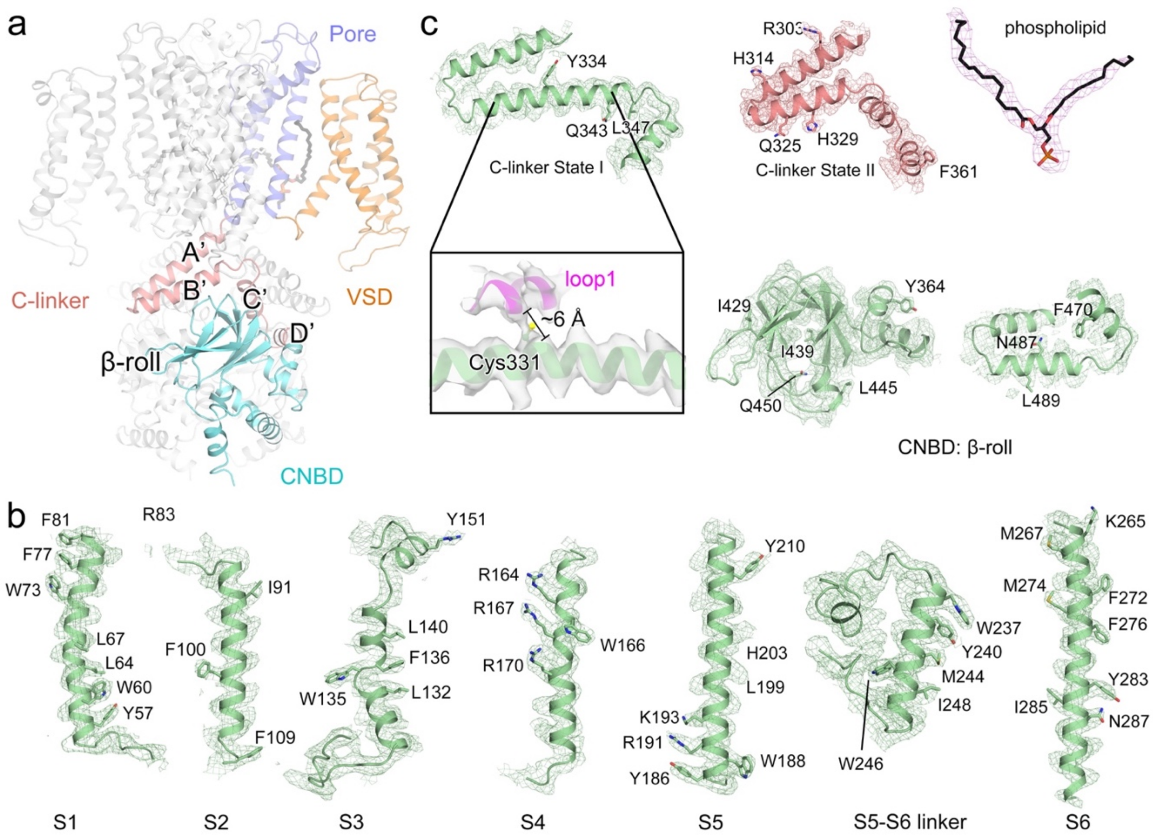

Supplementary Fig. 2 | The EM density maps for representative regions of AKT1.

a, An overall view of AKT1 with domain colored. **b-c,** The local EM density map for AKT1. The C-linkers in different states are shown individually. The density of loop1 has contact with Cys331 on C-linker. The contour level of the phospholipid is 5σ. The contour level of the EM density is 7σ.

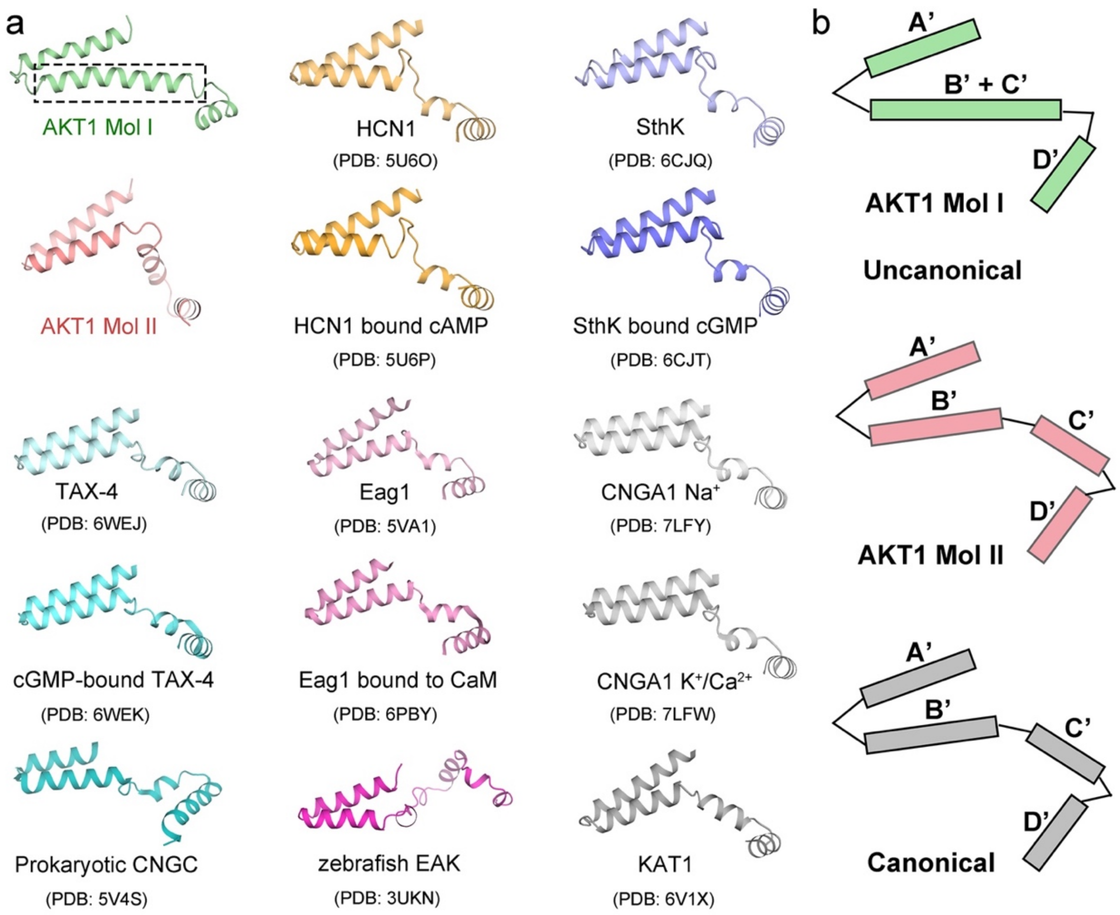

Supplementary Fig. 3 | Comparison of C-linkers of AKT1 and other ion channels.

a, The C-linkers of AKT1 and other ion channels as indicated. The C-linker of AKT1 displays two conformations. **b,** A cartoon to illustrate the uncanonical "straight" C-linker of AKT1 and the canonical conformations in other ion channels. The PDB codes are individually labelled.

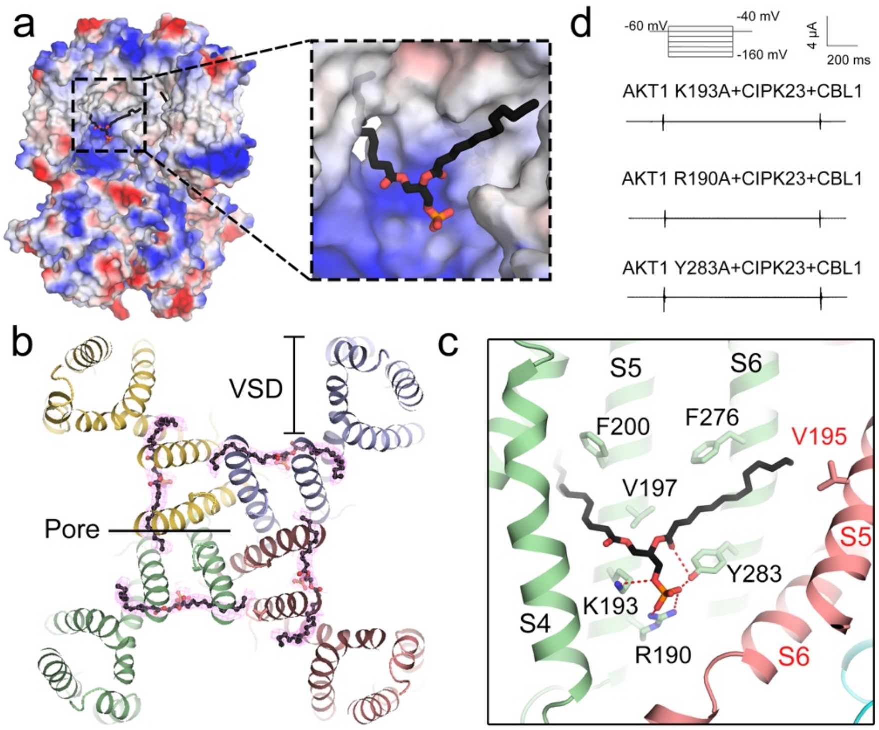

Supplementary Fig. 4 | Phospholipids binding site of AKT1. **a,** Surface potential representation of the phospholipid binding site. **b,** The four phospholipids are tightly associated with the core domains of AKT1. The densities of the phospholipids are colored in violet. **c,** The binding site of a phospholipid. The fatty acid tails insert into a hydrophobic groove. The polar head is coordinated by positively charged and polar residues including Arg190, Lys193, Tyr283. Point mutations on these three residues abrogate the K^+ mediated currents shown in **d**. Electrophysiological experiments were repeated using three different batches of oocytes with similar results. Source data are provided as a Source Data file.

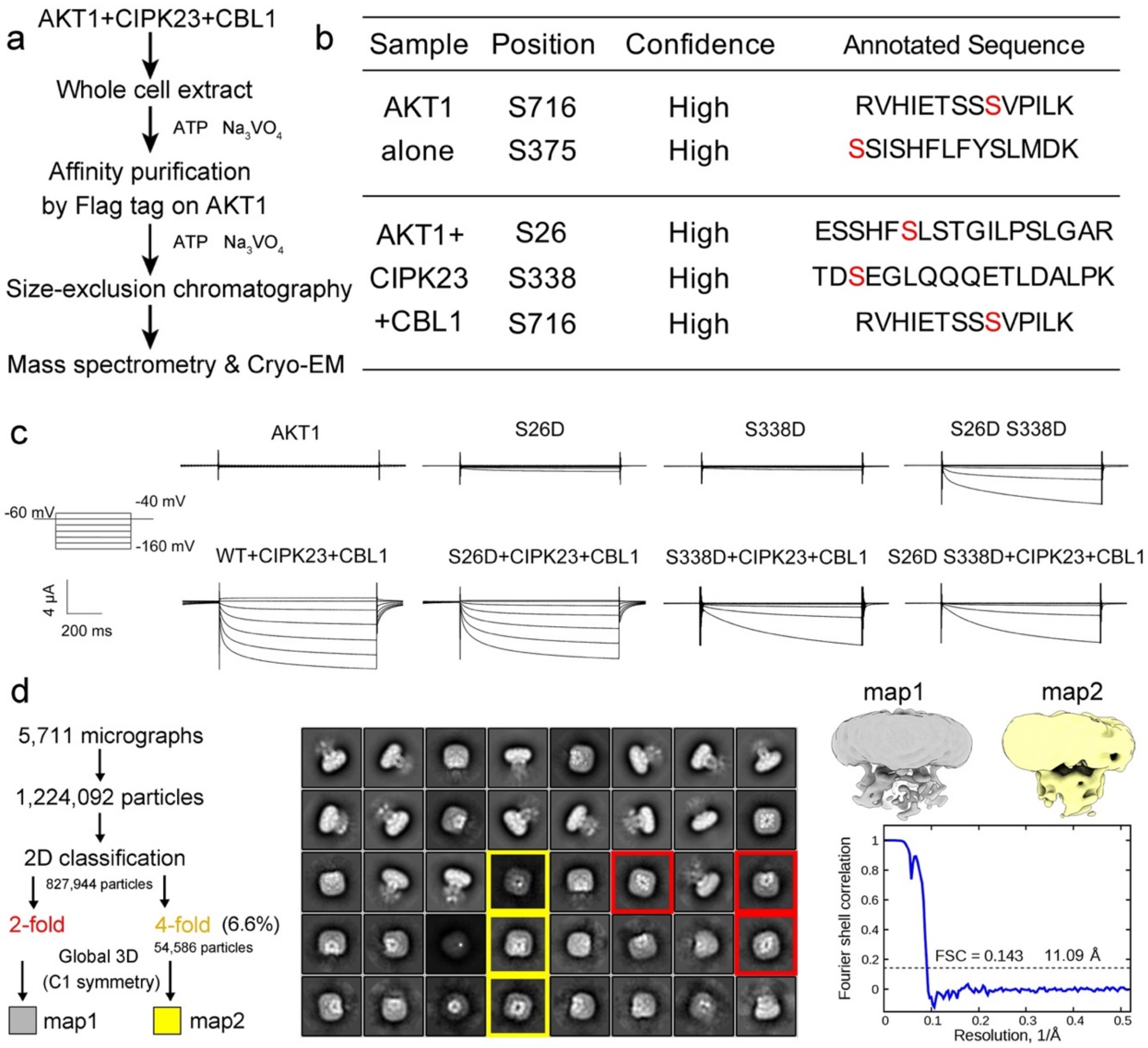

Supplementary Fig. 5 | Characterization of AKT1 co-expressed with CIPK23 and CBL1. **a,** A procedure for purification of AKT1 in the presence of kinases to mimic the phosphorylation cascade. **b,** Results of mass spectrometry for identification of additional phosphorylated peptides. **c,** Whole cell current trace of the phosphorylation mimetics recorded in *Xenopus* oocytes. Experiments were repeated using three different batches of oocytes with similar results. Source data are provided as a Source Data file.

d, Cryo-EM analysis of AKT1 co-expressed with kinase reveals two different structural arrangements.

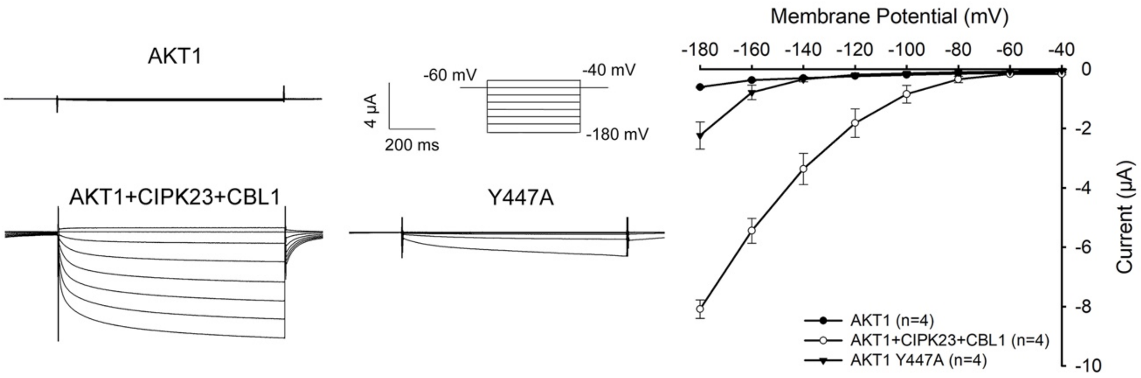

Supplementary Fig. 6 | Whole cell currents of the Tyr447Ala mutation. The I-V curve of steady-state currents in various oocytes is shown. Data are presented as means ± SEM. Specific *n* values are shown. Experiments were repeated three times with similar results. Source data are provided as a Source Data file.

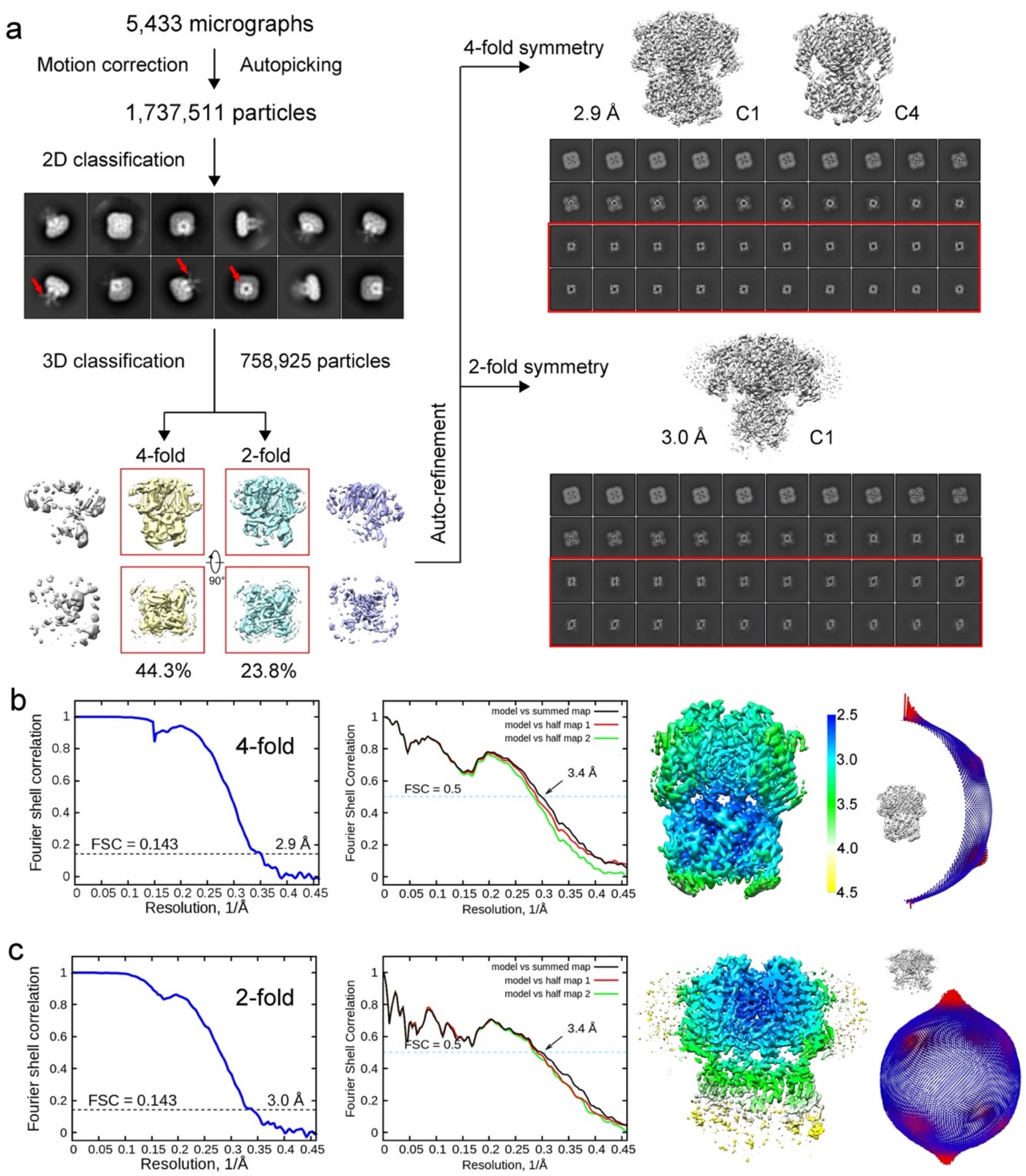

Supplementary Fig. 7 | Cryo-EM data processing and analysis for AKT1-Asp379Ala variant. **a,** A flowchart of the EM data processing. Two conformations of AKT1-Asp379Ala are identified. The cut-view of these two conformations are presented. **b-c,** The overall resolution of the reconstruction for AKT1-Asp379Ala is estimated to be 3.0 Å on the basis of the FSC 0.143 value (left). The FSC curves of the refined model versus

the map that it is refined against (black); of the model refined in the first of the two independent maps used for the FSC calculation versus that same map (red); and of the model refined in the first of the two independent maps versus the second independent map (green). The small difference between the red and green curves indicates that the refinement did not suffer from overfitting (middle). Angular distribution of the particles used for reconstruction of AKT1-Asp379Ala. Each cylinder represents one view and the height of the cylinder is proportional to the number of particles for that view (right).

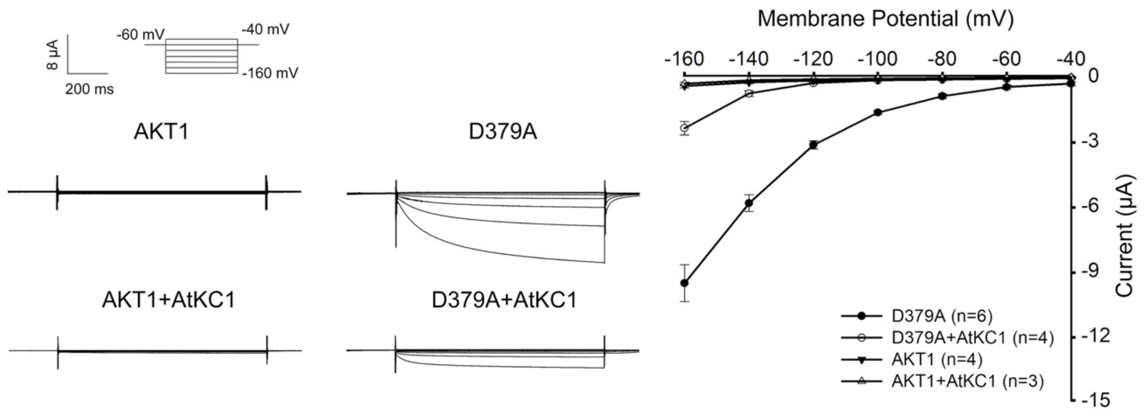

Supplementary Fig. 8 | Characterization of the regulatory role of AtKC1 for the constitutively-active mutant Asp379Ala. The I-V curve of steady-state currents in various oocytes is shown. Data are presented as means ± SEM. Specific *n* values are shown. Experiments were repeated using three different batches of oocytes with similar results. Source data are provided as a Source Data file.

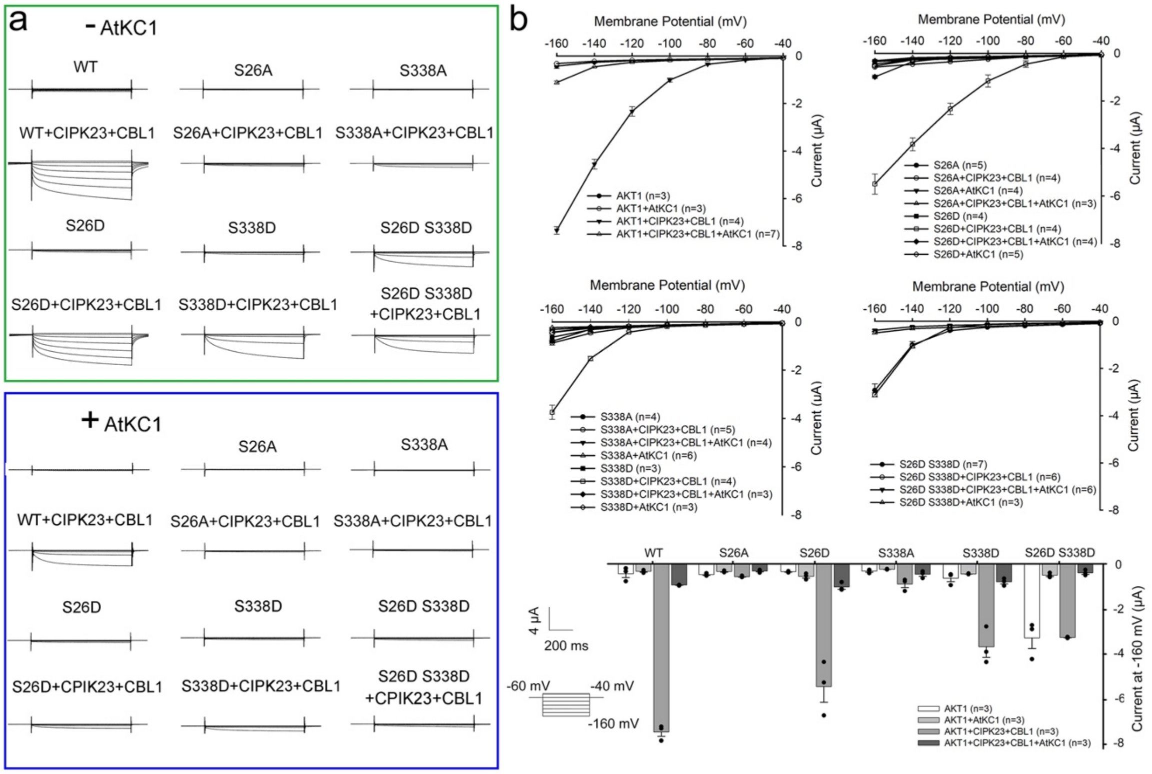

Supplementary Fig. 9 | AtKC1 inhibits the currents of AKT1 phosphor-mimetics.

a, The currents of AKT1 phosphor-mimetics in the absence (upper) or presence (lower) of AtKC1. **b,** The I-V curves of phosphor-mimetics at their steady-state (upper) and steady-state currents measured at -160mV (lower). Data are presented as means ± SEM. Specific *n* values are shown. Experiments were repeated three times with similar results. Source data are provided as a Source Data file.

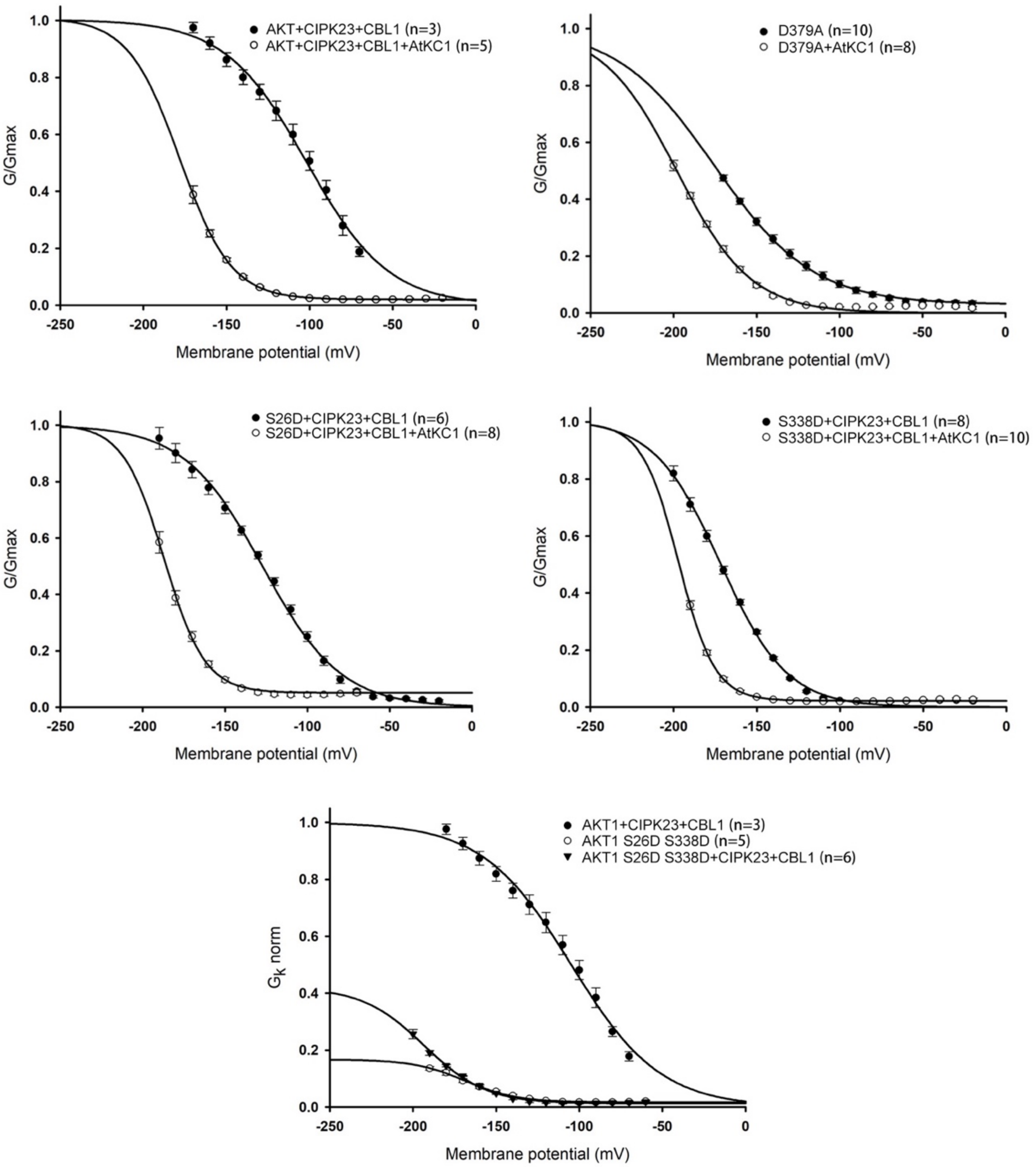

Supplementary Fig. 10 | Impact of the AKT1 mutants on the regulatory function of AtKC1 revealed by Boltzmann analysis. The voltage dependence (G/G_{max}) of inward K^+ currents from oocytes are shown. Though AtKC1 negatively shifts the activation potential for all tested mutants, the precise shift values are varying for different AKT1 mutants upon addition of AtKC1. The solid lines represented the best fits according to the Boltzmann functions: G/G_{max} (relative open probability) = $1/(1+\exp((V_m-V_{1/2})/S))$. G

(chord conductance) was calculated as $G=I/(V_m-E_K)$, where I is the steady-state current at voltage V_m, $V_{1/2}$ is the membrane potential at which the chord conductance is half-maximal and S is a slope factor. Data are presented as means ± SEM. Specific *n* values are shown. Experiments were repeated three times with similar results. Source data are provided as a Source Data file.

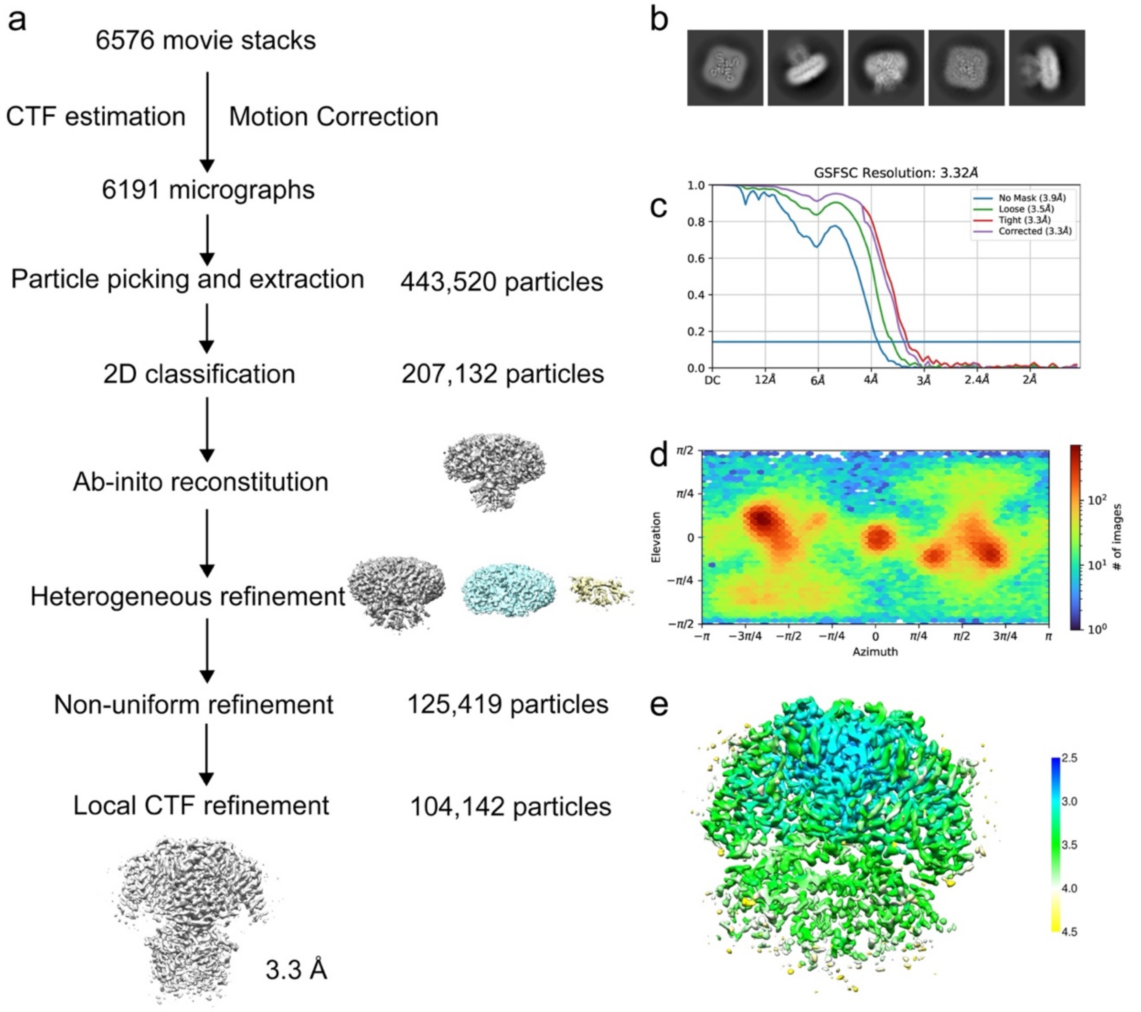

Supplementary Fig. 11 | Cryo-EM data processing and analysis for AKT1-AtKC1 complex. **a,** A flowchart of the EM data processing by Cryosparc[7]. **b-e,** Representative results of 2D classification. The overall resolution of the reconstruction for AKT1-AtKC1 is estimated to be 3.3 Å. Angular distribution of the particles used for reconstruction of AKT1-AtKC1. Local resolution estimation of AKT1-AtKC1.

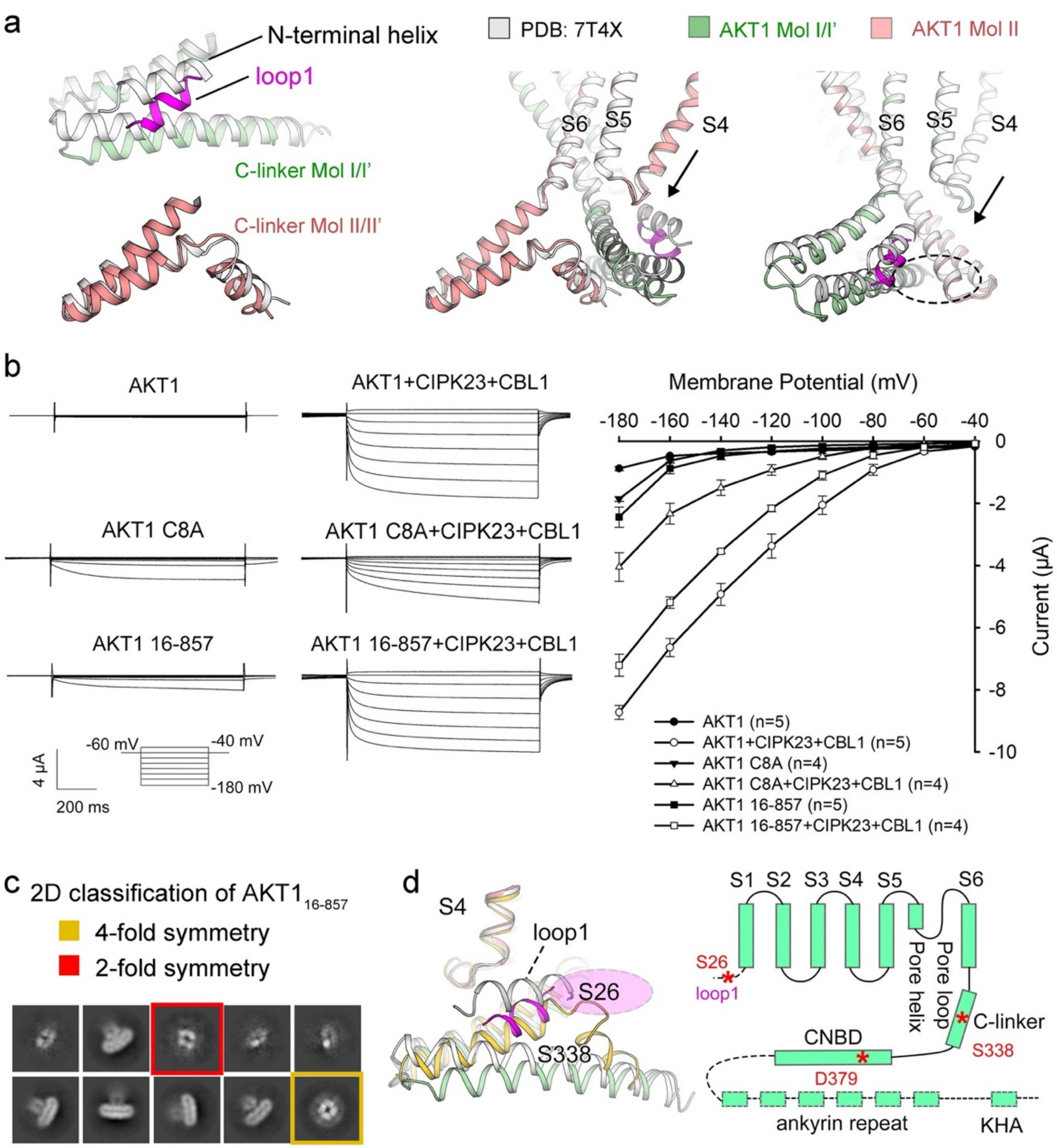

Supplementary Fig. 12 | Structural and functional characterization of loop1. a, Structural alignment of the loop1 and C-linker region between two independent studies (PDB: 7T4X[12]). **b,** Deletion of N-terminal residues (AKT1$_{16\text{-}857}$) or single mutant Cys8Ala alone leads to weak currents, which is enhanced by phosphorylation. The I-V curve of steady-state currents in various oocytes has been shown. Data are presented as means ± SEM. Specific *n* values are shown. Three biological repeats were performed

with similar results. Source data are provided as a Source Data file. **c,** 2D classification of $AKT1_{16\text{-}857}$. Deletion of N-terminal residues lead to 4-fold symmetric CPD. **d,** The model to illustrate the relationship between loop1, C-linker and the phosphorylation sites.

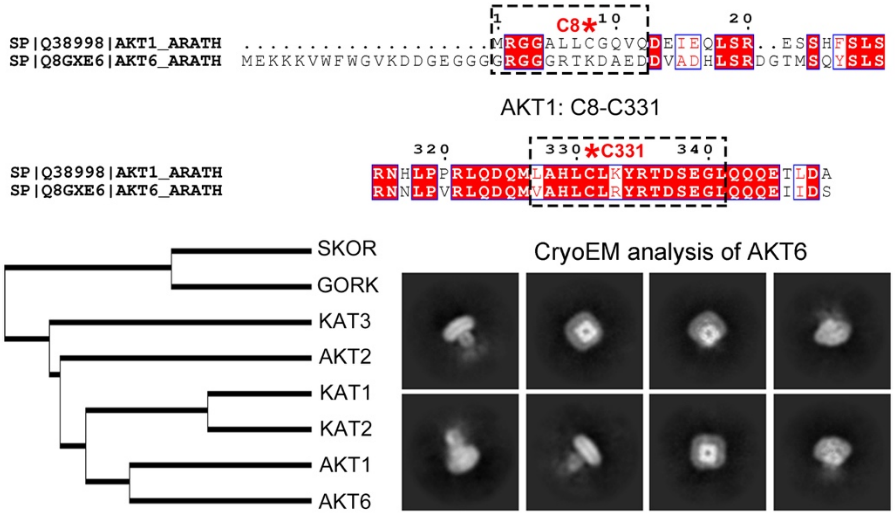

Supplementary Fig. 13 | Structural and functional differences between AKT1 and AKT6. Sequence alignment between AKT1 and AKT6 reveals differences at the N-terminal region. Different from AKT1, AKT6 exhibits a 4-fold symmetry conformation.

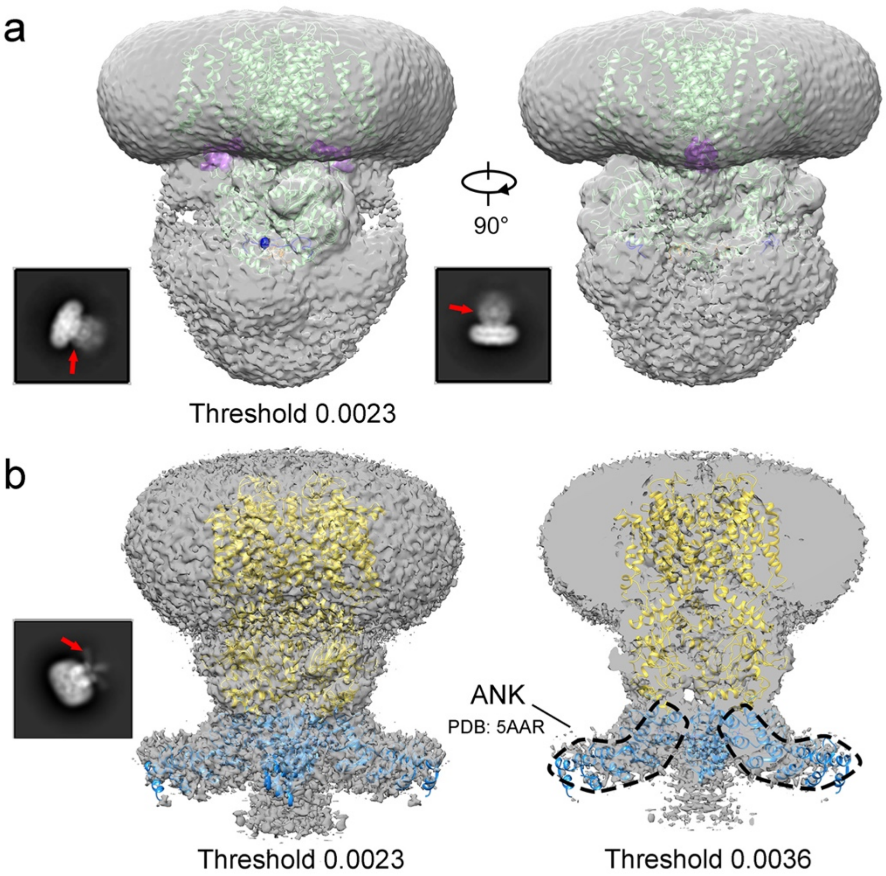

Supplementary Fig. 14 | Different conformations of ankyrin repeats in the low-pass filtered maps. Low contoured map of WT and the constitutively-active mutant are shown in **a-b.** The ANK domain can be docked into the map of the constitutively-active mutant.

Supplementary Table 1 | Cryo-EM data collection, refinement and validation statistics.

	WT AKT1 (EMDB-32769) (PDB 7WSW)	AKT1 variant (EMDB-31532) (PDB 7FCV)	AKT1-AtKC1 (EMDB-33467) (PDB 7XUF)
**Data collection and processing**			
Magnification	81,000	81,000	96,000
Voltage (kV)	300	300	300
Electron exposure (e–/Å^2)	50	50	51.07
Defocus range (μm)	-1.5 ~ -1.8	1.5 ~ -1.8	1.5 ~ -1.8
Pixel size (Å)	1.0825	1.0825	0.86
Symmetry imposed	C2	C4	C2
Initial particle images (no.)	349,544	1,737,511	443,520
Final particle images (no.)	181,714	323,861	104,142
Map resolution (Å)	3.4	3.0	3.3
FSC threshold	0.143	0.143	0.143
Map resolution range (Å)	2.5-3.5	2.5-3.5	2.5-3.5
**Refinement**			
Initial model used (PDB code)	-	-	-
Model resolution (Å)	3.6	3.4	3.4
FSC threshold	0.5	0.5	0.5
Model resolution range (Å)	3.6-256	3.4-256	3.4-256
Map sharpening *B* factor (Å^2)	-70	-118	-164.4
Model composition			
Non-hydrogen atoms	15,053	15,143	15,029
Protein residues	1,868	1,848	1,864
Ligands	7	7	3
B factors (Å^2)			
Protein	100.62	115.75	45.92
Ligand	77.93	85.61	28.56
R.m.s. deviations			
Bond lengths (Å)	0.002	0.010	0.004
Bond angles (°)	0.518	1.043	0.615
Validation			
MolProbity score	1.75	1.77	1.77
Clashscore	6.75	5.00	5.70
Poor rotamers (%)	0.00	0.00	0.06
Ramachandran plot			
Favored (%)	94.50	91.25	92.62
Allowed (%)	5.50	8.75	7.00
Disallowed (%)	0.00	0.00	0.38

Supplementary Table 2 | Channel property of various AKT1 channel complexes expressed in *Xenopus* oocytes.

Chanel	$V_{1/2}$ (mV)		Slope factor (mV)		Apparent gating valence z_g	
	**-AtKC1**	**+AtKC1**	**-AtKC1**	**+AtKC1**	**-AtKC1**	**+AtKC1**
AKT1 WT+CIPK23+CBL1	-101.7 ± 2.0	-178.2 ± 3.2	24.6 ± 1.3	15.5 ± 0.6	1.0 ± 0.05	0.65 ± 0.03
AKT1 D379A	-175.4 ± 4.2	-198.1 ± 10.2	29.6 ± 1.1	22.4 ± 2.4	1.2 ± 0.05	0.93 ± 0.1
AKT1 S26D+CIPK23+CBL1	-127.2 ± 1.2	-187.1 ± 3.7	23.8 ±0.9	12.5 ± 1.0	0.99 ± 0.04	0.52 ± 0.04
AKT1 S338D+CIPK23+CBL1	-170.8 ± 2.4	-197.1 ± 3.7	19.7 ± 1.1	10.9 ± 0.6	0.82 ± 0.05	0.45 ± 0.03
AKT1 S26D S338D	-168.5 ± 2.0	-	15.7 ± 1.0	-	0.65 ± 0.04	-
AKT1 S26D S338D+CIPK23+CBL1	-193.3 ± 5.8	-	18.6 ± 1.8	-	0.78 ± 0.08	-

Supplementary Table 3 | Primers used in this study.

Primer	Sequence (5' - 3')
AKT1-pFastBac1-F	GATTACAAGGATGACGATGACAAGGGTACCATGAGAGGCGGTGCT CTGCTGTGTGGT
AKT1-pFastBac1-R	GCATGCCTCGACCTATCACTCGAGTCAAGAGTCAGTGGCGAAGAT
AKT1-pCAG-F	GATGCAGGTACCGCCACCATGAGAGGCGGTGCT
AKT1-pCAG-R	AGCAAGCTTCTCGAGTCAAGAGTCAGTGGCGAA
CIPK23-pCAG-F	GGAGGTGGCGGACCCGGGATGGCTTCTCGAACA
CIPK23-pCAG-R	TCTGCTAGCAAGCTTCTCGAGTCATTATGTCGACTGTTTTGC
CBL1-pCAG-F	GAAGTTGATGCAGGTACCGCCACCATGGGCTGCTTCCAC
CBL1-pCAG-R	TCTGCTAGCAAGCTTCTCGAGTCATGTGGCAATCTCATC
AtKC1-pCAG-F	GGATCGGGAGGTTCGGCGTGGAGCCACCCGCAGTTCGAAAAAGGT AGCATGTCTACG
AtKC1-pCAG-R	AAGCTTCTCGACCTCGAGTCAGAAAATATATAA
AKT1 16-857-pCAG-F	GATGCAGGTACCGCCACCATGGAGCAGCTGTCC
AKT1 16-857-pCAG-R	GCTAGCAAGCTTCTCGAGTCAAGAGTCAGTGGC
AKT6-pCAG-F	GAAGTTGATGCAGGTACCATGGAGAAGAAGAAG
AKT6-pCAG-R	CTCGACTTATCACTCGAGTCAAGGATCCCTTGA
AKT1-pGEM-HE-F	GCAGATCAATTCCCCGGGATGAGAGGAGGGGCT
AKT1-pGEM-HE-R	GATCAAGCTTGCTCTAGATTAAGAATCAGTTGC
CIPK23-pGEM-HE-F	GCAGATCAATTCCCCGGGATGGCTTCTCGAACA
CIPK23-pGEM-HE-R	GATCAAGCTTGCTCTAGATTATGTCGACTGTTT
CBL1-pGEM-HE-F	GCAGATCAATTCCCCGGGATGGGCTGCTTCCAC
CBL1-pGEM-HE-R	GATCAAGCTTGCTCTAGATCATGTGGCAATCTC
AKT1 S26A-pGEM-HE-F	AGTAGTCATTTTGCTCTTTCTACTGGAATTTTA
AKT1 S26A-pGEM-HE-R	TCCAGTAGAAAGAGCAAAATGACTACTCTCTCT
AKT1 S338A-pGEM-HE-F	TACCGGACTGACGCTGAGGGTCTGCAACAGCAA
AKT1 S338A-pGEM-HE-R	TTGCAGACCCTCAGCGTCAGTCCGGTATTTCAA
AtKC1-pGEM-HE-F	GCAGATCAATTCCCCGGGATGTCTACGACGACT
AtKC1-pGEM-HE-R	GATCAAGCTTGCTCTAGATTAGAAAATATATAA
AKT1 S26D-pGEM-HE-F	AGTAGTCATTTTGATCTTTCTACTGGAATTTTA
AKT1 S26D-pGEM-HE-R	TCCAGTAGAAAGATCAAAATGACTACTCTCTCT
AKT1 S338D-pGEM-HE-F	TACCGGACTGACGATGAGGGTCTGCAACAGCAA
AKT1 S338D-pGEM-HE-R	TTGCAGACCCTCATCGTCAGTCCGGTATTTCAA
AKT1 D379A-pGEM-HE-F	GGAGTTAGCAACGCTCTCCTGTTCCAGCTCGTG
AKT1 D379A-pGEM-HE-R	CTGGAACAGGAGAGCGTTGCTAACTCCGCGAAA
AKT1 Y447A-pGEM-HE-F	GGGGTGTTATGCGCGAGACCACAACTGTTTACT

AKT1 Y447A-pGEM-HE-R	TTGCAGACCCTCATCGTCAGTCCGGTATTTCAA
AKT1 16-857-pGEM-HE-F	TTGGCAGATCAATTCCCCGGGATGGAACAGCTTTCGAGA
AKT1 16-857-pGEM-HE-R	GATCAAGCTTGCTCTAGATTAAGAATCAGTTGC
AKT1 C8A-pGEM-HE-F	ATCAATTCCCCGGGATGAGAGGAGGGGCTTTGTTAGCCGGACAAG TCCAAGATGAG
AKT1 C8A-pGEM-HE-R	TGCTCTAGAGAATTCTTAAGAATCAGTTGCAAA
AKT1 K193A-pGEM-HE-F	GTCCGATGCGCAGCACTCGTTTGTGTCACTCTT
AKT1 K193A-pGEM-HE-R	GACACAAACGAGTGCTGCGCATCGGACCCAAAA
AKT1 R190A-pGEM-HE-F	TACTTTTGGGTCGCATGCGCAAAACTCGTTTGT
AKT1 R190A-pGEM-HE-R	GAGTTTTGCGCATGCGACCCAAAAGTAGTTGAA
AKT1 Y283A-pGEM-HE-F	GGATTAACCGCTGCCTTGATTGGTAACATGACA
AKT1 Y283A-pGEM-HE-R	GTTACCAATCAAGGCAGCGGTTAATCCGAGGTT

References

1 Xu, J. *et al*. A protein kinase, interacting with two calcineurin B-like proteins, regulates K+ transporter AKT1 in Arabidopsis. *Cell* **125**, 1347-1360, doi:10.1016/j.cell.2006.06.011 (2006).

2 Lei, J. & Frank, J. Automated acquisition of cryo-electron micrographs for single particle reconstruction on an FEI Tecnai electron microscope. *J Struct Biol* **150**, 69-80, doi:10.1016/j.jsb.2005.01.002 (2005).

3 Zheng, S. Q. *et al*. MotionCor2: anisotropic correction of beam-induced motion for improved cryo-electron microscopy. *Nat Methods* **14**, 331-332, doi:10.1038/nmeth.4193 (2017).

4 Zhang, K. Gctf: Real-time CTF determination and correction. *J Struct Biol* **193**, 1-12, doi:10.1016/j.jsb.2015.11.003 (2016).

5 Grant, T. & Grigorieff, N. Measuring the optimal exposure for single particle cryo-EM using a 2.6 A reconstruction of rotavirus VP6. *Elife* **4**, e06980, doi:10.7554/eLife.06980 (2015).

6 Zivanov, J. *et al*. New tools for automated high-resolution cryo-EM structure determination in RELION-3. *Elife* **7**, doi:10.7554/eLife.42166 (2018).

7 Punjani, A., Rubinstein, J. L., Fleet, D. J. & Brubaker, M. A. cryoSPARC: algorithms for rapid unsupervised cryo-EM structure determination. *Nat Methods* **14**, 290-296, doi:10.1038/nmeth.4169 (2017).

8 Adams, P. D. *et al*. PHENIX: building new software for automated crystallographic structure determination. *Acta Crystallogr D Biol Crystallogr* **58**, 1948-1954 (2002).

9 Emsley, P. & Cowtan, K. Coot: model-building tools for molecular graphics. *Acta Crystallogr D Biol Crystallogr* **60**, 2126-2132, doi:10.1107/S0907444904019158 (2004).

10 Chen, V. B. *et al*. MolProbity: all-atom structure validation for macromolecular crystallography. *Acta Crystallogr D Biol Crystallogr* **66**, 12-21, doi:10.1107/S0907444909042073 (2010).

11 Pusch, M. *Expression and Analysis of Recombinant Ion Channels: From Structural Studies to Pharmacological Screening*. (Wiley Online Library, Weinheim, 2006).

12 Dickinson, M. S., Pourmal, S., Gupta, M., Bi, M. & Stroud, R. M. Symmetry Reduction in a Hyperpolarization-Activated Homotetrameric Ion Channel. *Biochemistry*, doi:10.1021/acs.biochem.1c00654 (2021).
