# Supplementary material for: Structural basis for the activity regulation of a potassium channel AKT1 from Arabidopsis
Source: Nat Commun. 2022 Sep 27;13:5682. doi: 10.1038/s41467-022-33420-8 (PMC9515098; doi:10.1038/s41467-022-33420-8)
Supplement: Supplementary file 2 — Reporting Summary [file 41467_2022_33420_MOESM2_ESM.pdf]

nature portfolio

Corresponding author(s): Guanghui Yang & Yi Wang

Last updated by author(s): Sep 2, 2022

Reporting Summary

Nature Portfolio wishes to improve the reproducibility of the work that we publish. This form provides structure for consistency and transparency in reporting. For further information on Nature Portfolio policies, see our Editorial Policies and the Editorial Policy Checklist.

Statistics

For all statistical analyses, confirm that the following items are present in the figure legend, table legend, main text, or Methods section.

n/a	Confirmed	
[x]	[ ]	The exact sample size (*n*) for each experimental group/condition, given as a discrete number and unit of measurement
[ ]	[x]	A statement on whether measurements were taken from distinct samples or whether the same sample was measured repeatedly
[x]	[ ]	The statistical test(s) used AND whether they are one- or two-sided<br>*Only common tests should be described solely by name; describe more complex techniques in the Methods section.*
[x]	[ ]	A description of all covariates tested
[x]	[ ]	A description of any assumptions or corrections, such as tests of normality and adjustment for multiple comparisons
[ ]	[x]	A full description of the statistical parameters including central tendency (e.g. means) or other basic estimates (e.g. regression coefficient) AND variation (e.g. standard deviation) or associated estimates of uncertainty (e.g. confidence intervals)
[x]	[ ]	For null hypothesis testing, the test statistic (e.g. *F*, *t*, *r*) with confidence intervals, effect sizes, degrees of freedom and *P* value noted<br>*Give P values as exact values whenever suitable.*
[x]	[ ]	For Bayesian analysis, information on the choice of priors and Markov chain Monte Carlo settings
[x]	[ ]	For hierarchical and complex designs, identification of the appropriate level for tests and full reporting of outcomes
[x]	[ ]	Estimates of effect sizes (e.g. Cohen's *d*, Pearson's *r*), indicating how they were calculated

Our web collection on statistics for biologists contains articles on many of the points above.

Software and code

Policy information about availability of computer code

Data collection	AutoEmation2; EPU
Data analysis	MotionCor2 1.2.1, CTFFIND 4.1.8, Gautomatch_v0.56_cu8.0, Relion3.1, Cryosparc-v3.1.1, Phenix1.19 , COOT0.9.5, UCSF-Chimera1.12, ChimeraX 1.2.5 Pymol 1.8.6.0, MolProbity (http://molprobity.manchester.ac.uk/), Clampex

For manuscripts utilizing custom algorithms or software that are central to the research but not yet described in published literature, software must be made available to editors and reviewers. We strongly encourage code deposition in a community repository (e.g. GitHub). See the Nature Portfolio guidelines for submitting code & software for further information.

Data

Policy information about availability of data

All manuscripts must include a data availability statement. This statement should provide the following information, where applicable:

- Accession codes, unique identifiers, or web links for publicly available datasets
- A description of any restrictions on data availability
- For clinical datasets or third party data, please ensure that the statement adheres to our policy

The 3D cryo-EM density maps have been deposited in the Electron Microscopy Data Bank (EMDB) under the accession code EMD-32769 [https://www.ebi.ac.uk/pdbe/entry/emdb/EMD-32769] (AKT1 WT), EMD-31532 [https://www.ebi.ac.uk/pdbe/entry/emdb/EMD-31532] (AKT1 Asp379Ala, constitutively-active mutant), and EMD-33467 [https://www.ebi.ac.uk/pdbe/entry/emdb/EMD-33467] (AKT1-AtKC1 complex). The atomic coordinates for the corresponding model have been deposited in the Protein Data Bank (PDB) under the accession code 7WSW [http://doi.org/10.2210/pdb7WSW/pdb] (AKT1 WT), 7FCV [http://doi.org/10.2210/

pdb7FCV/pdb] (AKT1 Asp379Ala, constitutively-active mutant), and 7XUF [http://doi.org/10.2210/pdb7XUF/pdb] (AKT1-AtKC1 complex). Source data underlying Figures 1a, 3c, 4c-d, 6a, and Supplementary Figures 4d, 5c, 6, 8, 9, 10, 12b are provided as a Source Data file. Source data are provided with this paper.

Human research participants

Policy information about studies involving human research participants and Sex and Gender in Research.

Reporting on sex and gender	n/a
Population characteristics	n/a
Recruitment	n/a
Ethics oversight	n/a

Note that full information on the approval of the study protocol must also be provided in the manuscript.

Field-specific reporting

Please select the one below that is the best fit for your research. If you are not sure, read the appropriate sections before making your selection.

☒ Life sciences ☐ Behavioural & social sciences ☐ Ecological, evolutionary & environmental sciences

For a reference copy of the document with all sections, see nature.com/documents/nr-reporting-summary-flat.pdf

Life sciences study design

All studies must disclose on these points even when the disclosure is negative.

Sample size	No statistical methods were used to predetermine sample size. performed. The data size for cryo-EM was determined by the availability of the microscope time and the particle density on the grids. Sufficient cryo-EM data were collected to achieve the reported resolution of map, which is sufficient for model building. For cryoEM experiments, particle number was chosen to maximize map quality and resolution. For electrophysiology experiments, cell number was chosen based on convention in the field (at least 4). This was deemed to be sufficient to determine mean and standard error of the mean, allowing for comparison between different mutants.
Data exclusions	For cryoEM experiments, particles were excluded if they did not improve map quality. This is standard practice for cryoEM structure determination. For electrophysiological experiments, recordings were excluded from analysis if leak or endogenous currents prevented analysis.This is standard practice in electrophysiology.
Replication	Each experiment was reproduced at least two times on separate occasions. Experimental findings were reliably reproduced. During the structure determination, the quality of the density map was improved through 3D classification and local refinement as indicated in the method. The PDB model was manually refined in COOT and automatically refined in Phenix for several times. For electrophysiological results, experiments were repeated using three different batches of oocytes, ensuring reproducibility.
Randomization	Randomization was not employed, as is standard for structural and electrophysiological work. Randomization was not necessary as the independent variables to be tested were sufficient for the functional interpretations within this study. i.e. WT vs mutant vs control conditions.
Blinding	Blinding is not necessary or valid for the purposes of structural determination. Blinding was not employed, as is standard for structural and electrophysiological work. For functional analysis, blinding was not necessary due to the quantitative nature of the experiment. All experimental data acquired in included in our statistical analysis.

Reporting for specific materials, systems and methods

We require information from authors about some types of materials, experimental systems and methods used in many studies. Here, indicate whether each material, system or method listed is relevant to your study. If you are not sure if a list item applies to your research, read the appropriate section before selecting a response.

Materials & experimental systems

n/a	Involved in the study	
☒	☐	Antibodies
☐	☒	Eukaryotic cell lines
☒	☐	Palaeontology and archaeology
☐	☒	Animals and other organisms
☒	☐	Clinical data
☒	☐	Dual use research of concern

Methods

n/a	Involved in the study	
☒	☐	ChIP-seq
☒	☐	Flow cytometry
☒	☐	MRI-based neuroimaging

Eukaryotic cell lines

Policy information about cell lines and Sex and Gender in Research

Cell line source(s)	sf9 (Invitrogen); HEK293F (Invitrogen)
Authentication	No further authentication was performed for commercially available cell lines.
Mycoplasma contamination	not tested
Commonly misidentified lines (See ICLAC register)	not used

Animals and other research organisms

Policy information about studies involving animals; ARRIVE guidelines recommended for reporting animal research, and Sex and Gender in Research

Laboratory animals	Xenopus laevis
Wild animals	The study did not involve wild animals.
Reporting on sex	The study is not based on sex.
Field-collected samples	The study did not involve samples collected from the field.
Ethics oversight	Animal studies were conducted in accordance with the ethical guidelines of Ministry of Agriculture (Beijing, China). The animal experiments conformed to the guidelines and regulatory standards of the Institutional Animal Care and Use Committee of China Agricultural University, no. AW20902202-3-1.

Note that full information on the approval of the study protocol must also be provided in the manuscript.
